# Evolutionary and functional analysis of ARF and Aux/IAA gene families reveals their roles in sugar metabolism and watermelon domestication

**DOI:** 10.3389/fpls.2026.1868206

**Published:** 2026-06-17

**Authors:** Shao Zhengwei, Fikru Tamiru Kenea, Chen Gang, Yang Dongdong, Chen Mengli, Zhang Yushi, Lu Xuqiang, Zhu Hongju, He Nan, Liu Wenge

**Affiliations:** 1 Henan Joint International Research Laboratory of South Asian Fruits and Cucurbits, Zhengzhou Fruit Research Institute, Chinese Academy of Agricultural Sciences, Zhengzhou, Henan, China; 2 Innovation and Utilization Team of Tropical Melon and Fruit Germplasm Resources, National Nanfan Research Institute of Chinese Academy of Agricultural Sciences, Sanya, Henan, China; 3 Department of Horticulture, College of Agriculture and Natural Resources, Dilla University, Dilla, Ethiopia; 4 Zhongyuan Research Center, Chinese Academy of Agricultural Sciences, Xinxiang, Henan, China

**Keywords:** auxin, *Citrullus lanatus*, comparative genomics, fructose, glucose

## Abstract

Auxin signaling, mediated by auxin response factors (ARFs) and Aux/IAA proteins, is central to plant development; however, its role in regulating fruit quality traits-particularly soluble sugar and organic acid metabolism-remains largely unexplored in Cucurbitaceae crops. To address this gap, we integrated comparative genomics, metabolomics, and transcriptomics to elucidate the evolutionary conservation and functional specialization of *ARF* and *Aux/IAA* gene families in cucurbit fruit quality regulation. We identified 157 *ARF* and 275 *Aux/IAA* genes across eight cucurbit species, revealing extensive evolutionary conservation under predominant purifying selection (Ka/Ks < 0.6). Despite this constraint, domestication-driven functional divergence was evident: comparative metabolomic and population genomic analyses of wild watermelon ancestors (*Citrullus amarus* and *C. mucosospermus*) and cultivated accessions showed that glucose and fructose contents increased sharply during the *C. amarus* to *C. mucosospermus* transition, whereas sucrose accumulation rose later during the shift from seed-type to landrace watermelons. Four candidate genes (*ClaARF4*, *ClaARF15*, *ClaIAA4*, and *ClaIAA22*) exhibited significant allelic differentiation during this early sugar-accumulation transition and remained stably fixed in subsequent domestication stages, directly linking evolutionary signatures with functional outcomes. Exogenous auxin treatment increased glucose and fructose by 23–35% while reducing malate by 18%, confirming that auxin signaling reprograms carbon partitioning toward hexose accumulation. Transcriptome profiling revealed that most genes peaked during early fruit development (10–22 days after pollination), coinciding with metabolic shifts in sugar and organic acid accumulation. Promoter analysis uncovered conserved cis-regulatory elements associated with fruit development across watermelon, cucumber, and melon, suggesting shared regulatory logic. Collectively, this study provides the first integrated evidence that *ARF* and *Aux/IAA* gene families coordinate fruit quality metabolism in cucurbits through evolutionarily conserved regulatory architecture with lineage-specific functional adaptations. These findings establish a molecular framework for cucurbit quality improvement, offering candidate targets for marker-assisted selection or genome editing to enhance fruit sweetness and flavor.

## Introduction

Auxin, the first discovered phytohormone, plays pivotal roles in plant growth, development, quality formation, and stress adaptation through the auxin response factor (ARF) and IAA gene signaling module (Moeen-Ud-Din et al., 2023; Hammes and Pedersen, 2024; Xing et al., 2024). This system operates as a molecular dimerization switch, in which, at low auxin concentrations, the C-terminal dimerization domain (CTD) of ARF proteins binds to IAA proteins and suppresses ARF transcriptional activity, whereas auxin accumulation triggers ubiquitination and subsequent 26S proteasome-mediated degradation of CTD-bound IAA proteins, thereby releasing ARF transcriptional activity (Weijers et al., 2005; Die et al., 2018; Li et al., 2023). Furthermore, the variable middle region (MR) of ARF proteins modulates CTD interactions, influencing both ARF subcellular localization and transcriptional regulatory capacity (Powers et al., 2019). For instance, *MdIAA2* in apple represses auxin-responsive genes through interaction with ARFs, leading to reduced fruit size; *MoIAA13* negatively regulates shoot regeneration in drumstick tree, while *DgIAA21* impairs drought adaptation in orchardgrass (Bu et al., 2022; Yang et al., 2022; Wang et al., 2023). Together, these studies highlight coordinated ARF-IAA interactions as central to auxin signaling. Recent studies have demonstrated that ARF and Aux/IAA genes are involved not only in plant architecture and stress adaptation but also in fruit quality formation and metabolite accumulation (Jin et al., 2024). In tomato, *SlARF4* and *SlARF6* regulate sugar accumulation and fruit metabolism during ripening (Sagar et al., 2013; Yuan et al., 2019). Similarly, auxin-responsive genes have been associated with sugar metabolism and fruit quality regulation in citrus and other fruit crops (Durán-Soria et al., 2020). Despite these advances, the relationship between auxin signaling and fruit quality in Cucurbitaceae crops remains insufficiently understood, particularly during domestication and fruit development.

In the Cucurbitaceae family, recent genome-wide studies have mapped these gene families, though research has largely focused on fruit morphogenesis or stress responses. In cucumber (*Cucumis sativus*), 18 ARF and 29 IAA genes have been linked to various vegetative and reproductive developmental processes (Gan et al., 2013; Zhang et al., 2025). Similarly, in pointed gourd (*Trichosanthes dioica* Roxb.), 48 ARF transcripts and 37 IAA genes, whose interaction complexes are suggested to regulate fruit growth before fertilization, have been identified (Saurabh et al., 2022). In melon (*Cucumis melo*) and bottle gourd (*Lagenaria siceraria*), ARF and IAA have been reported as regulators of early fruit expansion and maturation (Wu et al., 2020; He et al., 2024). Moreover, 33 ARF genes identified in zucchini (*Cucurbita pepo*) have been demonstrated to confer enhanced stress tolerance when heterologously expressed in *Arabidopsis*, further highlighting the functional diversity of ARF genes across cucurbit crops (Zhang et al., 2024).

Watermelon (*Citrullus lanatus*) is one of the most economically important fruit crops worldwide, and its commercial quality is strongly influenced by soluble sugar, including fructose, glucose, sucrose, and organic acid content, which are key determinants of consumer preference. Several candidate genes regulating these metabolites have been uncovered, though their polygenic character demands further investigation of candidate genes (Kenea et al., 2025). Auxin serves as a core phytohormone that orchestrates fundamental processes such as cell elongation, organ differentiation, and tropic responses across plant species. In cucurbits, recent studies have further elucidated how this hormone signaling pathway is specifically recruited to regulate tendril morphogenesis, parthenocarpy, and sex determination, thereby linking general auxin biology with cucurbit−specific developmental innovations (Nie et al., 2025; Han et al., 2026). Although Aux/IAA (IAA) family genes have been linked to fruit ripening and firmness, as evidenced by the delayed ripening observed in *ClIAA16* knockout mutants (Anees et al., 2023; Hu et al., 2023), direct evidence connecting auxin signaling components, including ARF and IAA genes, to sugar and organic acid metabolism remains limited.

Therefore, this study addressed the knowledge gap regarding the evolutionary and functional roles of *ARF* and *Aux/IAA* gene families in regulating fruit quality metabolism in cucurbits, particularly sugar and organic acid metabolism during watermelon fruit domestication and fruit development. We hypothesized that specific *ARF* and *Aux/IAA* genes underwent domestication associated functional differentiation and contribute to metabolic transitions during evolution and early fruit development through auxin signaling. Accordingly, the objective of this study was to systematically characterize *ARF* and *Aux/IAA* gene families during domestication and fruit development, and to elucidate their potential roles in auxin-mediated fruit quality formation. To achieve this, we employed an integrated approach combining molecular biology, bioinformatics, and plant physiological analyses across eight cucurbit crops. Our comprehensive investigation identified 157 ARF and 275 IAA genes, which were systematically characterized through detailed examination of gene architectures, phylogenetic relationships, syntenic conservation, cis-regulatory elements, and expression profiles. We further evaluated the effects of exogenous auxin on both transcriptional regulation of these genes and metabolic changes in sugar and organic acid composition during watermelon fruit development. Importantly, through domestication analysis, we delineated the evolutionary pathway of sugar accumulation in watermelon and identified a crucial regulatory gene controlling this metabolic process. The objective of this study was to systematically uncover how *ARF* and *Aux/IAA* genes mediate auxin signaling to regulate fruit quality metabolites in cucurbits, with a specific focus on watermelon. We hypothesized that exogenous auxin influences the transcriptional dynamics of these genes, thereby modulating sugar and organic acid accumulation during fruit development, and that key regulators in this pathway have been selected during domestication to enhance fruit sweetness. This work provides the first systematic evidence for ARF and IAA-mediated regulation of fruit quality metabolites in watermelon, offering fundamental insights into auxin signaling mechanisms and practical targets for molecular breeding of improved fruit quality traits.

## Materials and methods

### Genome-wide identification and chromosomal localization of ARF and IAA genes in eight cucurbit crops

In this study, we performed systematic identification of ARF and IAA gene family members from eight cucurbit crops: watermelon (*Citrullus lanatus*), bottle gourd (*Lagenaria siceraria*), pumpkin (*Cucurbita moschata*), chayote (*Sechium edule*), bitter melon (*Momordica charantia*), wax gourd (*Benincasa hispida*), cucumber (*Cucumis sativus*), and melon (*Cucumis melo*). All reference genome data were obtained from the Cucurbit Genomics Database (http://cucurbitgenomics.org/v2), with the dataset accessed and downloaded on July 18, 2023.

Gene identification was performed using HMMER software (v3.3.2; http://hmmer.org/) with hidden Markov model (HMM) profiles of characteristic domains obtained from the PFAM database (https://pfam.xfam.org/), including the B3 (PF02362), ARF (PF06507), and IAA (PF02309) domains. HMMER searches were conducted with an E-value threshold of <0.00001 to ensure high stringency. Candidate genes identified by HMMER were subsequently validated using the SMART database (http://smart.embl-heidelberg.de/) to confirm the presence of complete ARF or AUX/IAA domains, serving as the final criterion for gene retention. Only genes containing verified complete domains were retained for downstream analysis. Finally, using chromosomal position information from genome annotation files, all identified ARF and IAA genes were precisely mapped to their respective chromosomes, providing a comprehensive foundation for subsequent gene family analyses.

### Phylogenetic analysis of ARF and IAA genes in eight cucurbit crops

To systematically investigate the evolutionary relationships of ARF and IAA genes among eight cucurbit crops, we implemented the following analytical pipeline. First, multiple sequence alignment (MSA) of the identified full-length protein sequences was performed using the ClustalW algorithm integrated in MEGAX with default parameters (gap open=-2.9, gap extend= 0, hydrophobicity multiplier=1.2) to ensure alignment accuracy. Subsequently, the aligned sequences were subjected to phylogenetic analysis using MEGAX.0 (Kumar et al., 2016) with the JTT+G model (Jones-Taylor-Thornton substitution matrix with gamma-distributed rate variation among sites, 5 discrete gamma categories). Initial trees for ML analysis were obtained using the Neighbor-Joining (NJ) method, and tree topology was optimized using the Nearest-Neighbor-Interchange (NNI) heuristic algorithm. Bootstrap analysis with 1,000 replicates was conducted to assess branch support and statistical confidence, with values ≥70% considered indicative of robust support. For enhanced visualization, the phylogenetic trees were refined using Evolview online tools (https://www.evolgenius.info/evolview/) and the ggtree R package, incorporating branch coloring and subfamily classification annotations. Furthermore, a divergence time tree for the eight cucurbit crops was retrieved from the Timetree database (https://timetree.org/), which provides peer-reviewed molecular clock-based evolutionary timelines.

### Identification of homologous genes and Ka/Ks analysis in eight cucurbit crops

To systematically analyze the evolutionary relationships of ARF and IAA gene families, whole-genome synteny analysis was performed using McScanX (Wang et al., 2012). Orthologous gene pairs among the eight cucurbit crops and paralogous gene pairs within each species were identified for both gene families using default parameters. The homologous gene pairs were subsequently integrated using custom Python scripts and visualized as whole-genome synteny maps with NGenomeSyn (He et al., 2023).

To evaluate selection pressure on homologous genes, evolutionary parameters were calculated using KaKs_Calculator 2.0 (Wang et al., 2010), including the nonsynonymous substitution rate (Ka), synonymous substitution rate (Ks), and their ratio (Ka/Ks). The Ka/Ks ratio served as an indicator of selection pressure, where values >1 suggest positive selection, = 1 indicate neutral selection, and <1 signify purifying selection (Roth and Liberles, 2006).

### Analysis of cis-acting elements in the promoter regions of ARF and IAA genes in watermelon

To elucidate the transcriptional regulation mechanisms of ARF and IAA genes, 2 kb genomic sequences upstream of the initiation codon (ATG) were extracted as promoter regions. Based on comparative analysis of the watermelon pan-genome data (http://www.watermelondb.cn/), *ClaARF7* (*Cla97C03G057760.1*) was mapped to *ClG42_03g0065100.1*, and the missing promoter sequence was subsequently retrieved for downstream analyses. The promoter sequences were comprehensively scanned for cis-acting elements using the PlantCARE database (http://bioinformatics.psb.ugent.be/webtools/plantcare/html/), followed by functional annotation. The results were systematically analyzed to quantify the occurrence and distribution patterns of the identified cis-acting elements.

### Expression analysis of ARF and IAA genes in fruits of watermelon, melon, and cucumber

The transcriptomic profiles of watermelon fruits were obtained from our previous study (2018) examining different developmental stages of cultivars ‘203Z’ and ‘SW’ (Gao et al., 2018). Cucumber and melon fruit expression data were retrieved from the NCBI BioProject database (accession numbers PRJNA707246 and PRJNA796042, respectively), and corresponding FPKM (Fragments Per Kilobase of transcript per Million mapped reads) values were downloaded from the Cucurbit Genomics Database. Expression matrices were constructed for ARF and IAA gene family members in each species using R project. To ensure robust and reproducible gene expression analysis, low-expressed genes were filtered using the filterByExpr() function from the edgeR package.

### Plant materials and exogenous IAA treatment and analysis of soluble sugars and organic acids during watermelon fruit development

In this study, the red-fleshed large-fruited watermelon cultivar ‘HeiFeiCuiXuanHua’, bred by our research team, was selected as the experimental material (preserved at the Henan Joint International Research Laboratory of South Asian Fruits and Cucurbits, Zhengzhou Fruit Research Institute, Chinese Academy of Agricultural Sciences). Plant and flesh characteristics are shown in **Supplementary figure 1**. The experiment was arranged in a randomized complete block design (RCBD) with three biological replications, each replication comprising 12 plants per treatment. Seeds were sown in October 2023 at the Nanbin Farm experimental base of the Zhengzhou Fruit Research Institute, Chinese Academy of Agricultural Sciences (Sanya, Hainan, China; 18.36°N, 109.19°E). Based on preliminary experiments, exogenous indole-3-acetic acid (IAA) treatment at 100 μM was determined to be optimal for investigating auxin-responsive gene expression during fruit development. Following self-pollination, IAA treatment was initiated at 10 days after pollination (DAP) by foliar application of 100 μM IAA solution to the fruit surface and the three leaves proximal to the peduncle until runoff. Control plants received ddH_2_O treatment under identical conditions. Treatments were reapplied at 6-day intervals, and fruit samples were collected at 16, 22, 28, and 34 DAP.

According to the sampling protocol established by Ren et al. (2018), at each sampling point, a 1 cm³ cube of flesh from the fruit center was excised. One portion was immediately juiced for soluble sugar and organic acid analysis, while the other was flash-frozen in liquid nitrogen and stored at −80 °C for subsequent use. Three biological replicates were collected for each treatment. Glucose (G0504W), sucrose (G0531W), fructose (G0530W), citric acid (G0837W), and malic acid (G0862W48) were quantified using colorimetric assays with commercially available kits (Suzhou Grace Biotechnology, China), following the manufacturer’s protocols. Standard curves (0–100 μg/mL, R² > 0.99) were prepared using kit-supplied standards. Absorbance was measured using Varioskan LUX (Thermo Fisher Scientific, America).The contents of soluble sugars and organic acids contents were expressed as mg/g FW. Three biological replicates (each from three pooled fruits) were analyzed with three technical replicates each.

### Effects of exogenous IAA treatment on the expression regulation of ARF and IAA genes in watermelon

Gene-specific primers for nine candidate genes were designed using Primer3 (Untergasser et al., 2012), primer sequences provided in **Supplementary Table S2**) Total RNA was extracted from frozen fruit tissues sampled at different developmental stages and treatments using the EASYspin Universal Plant RNA Kit (Labhelper, China), followed by cDNA synthesis with TRUEscript RT MasterMix (Labhelper). Quantitative real-time PCR (qPCR) was performed using SYBR qPCR Master Mix (Tolobio, China) in a 20 µL reaction mixture containing 10 µL SYBR Green Master Mix, 1 µL primer mix (0.5 µL each of forward and reverse primers), 1 µL cDNA template, and nuclease-free water.

The thermal cycling protocol consisted of an initial denaturation at 95 °C for 5 min, followed by 40 cycles of 95 °C for 10 sec and 60 °C for 30 sec. The watermelon actin gene *ClaACTIN* (*Cla97C02G026960.1*) served as the internal reference for normalization. Relative gene expression levels were calculated using the 2^−ΔΔCt^ method to assess differential expression patterns across developmental stages and treatments (Livak and Schmittgen, 2001).

### Statistical analysis

All data are expressed as mean ± standard error (SE) from three biological replicates. Statistical analyses were conducted using R project with the emmeans and multcomp packages. A two-way analysis of variance (ANOVA) was performed to assess the main effects of treatment (IAA vs. control) and days after pollination (DAP), as well as their interaction effects, on sugar content, organic acid levels, and gene expression. When significant differences were detected (P < 0.05), *post hoc* pairwise comparisons among all treatment × DAP combinations were conducted using Tukey’s Honestly Significant Difference (HSD) test. Results are presented using compact letter display (CLD), where groups sharing the same letter are not significantly different at α = 0.05. Exact P values from ANOVA are reported in supplementary tables. Error bars in all figures represent standard error (SE).

### Domestication analysis of soluble sugars and key regulatory genes in watermelon fruits

Based on our previous research and metabolomic data (Yuan et al., 2023), we quantified the glucose, fructose, and sucrose contents across 211 watermelon accessions. These materials were classified into six distinct populations: *C. colocynthis* (CC, 9), *C. amarus* (CA, 5), *C. mucosospermus* (CM, 18), *C. lanatus* edible seed (CL_Es, 12), *C. lanatus* landrace (CL_Lr, 53), and *C. lanatus* improved (CL_Im, 114). Detailed information on population sample sizes and geographic origins is provided in **Supplementary Table 3**.

To elucidate the population structure and genetic diversity of these 211 accessions, we conducted systematic analyses using published SNP datasets (Guo et al., 2019). SNPs located within ARF and IAA genes were extracted from the whole-genome SNP data. Population structure was then analyzed using STRUCTURE software v2.3.4 (Pritchard et al., 2000) based on allele frequencies. For each K value ranging from 2 to 15, 20 independent runs were performed. The optimal number of populations was determined using the ΔK method, and individual population assignments (K = 2-8) were established through 10,000 iterations of SNP analysis. To investigate genetic variation patterns of candidate genes across populations, we identified SNPs in ARF and IAA genes, focusing on nonsynonymous mutations in coding regions. Statistical significance of allele frequency differences between wild and cultivated populations was assessed using Fisher’s exact test for each candidate SNP. P-values were adjusted for multiple testing using the false discovery rate (FDR) method (Benjamini and Hochberg, 1995), with a significance threshold of FDR < 0.05. The genotype distribution and allele frequencies of these variants were systematically analyzed across populations, and the results were visualized as stacked percentage bar charts using Origin 2021. To further characterize the genetic architecture of candidate genes under selection, haplotype analysis was performed for ARF and IAA genes showing significant associations with sugar content. Nonsynonymous SNPs within the coding regions of each candidate gene were extracted from the whole-genome SNP dataset and used for haplotype construction. Haplotype analysis was conducted using the geneHapR package v1.2.3 (Zhang et al., 2023). Haplotype frequencies across the six populations (CC, CA, CM, CL_Es, CL_Lr, and CL_Im) were calculated and visualized to illustrate the domestication trajectory from wild to cultivated watermelon.

## Results

### Identification and classification of ARF and IAA genes in eight cucurbit crops

**Figure 1:**
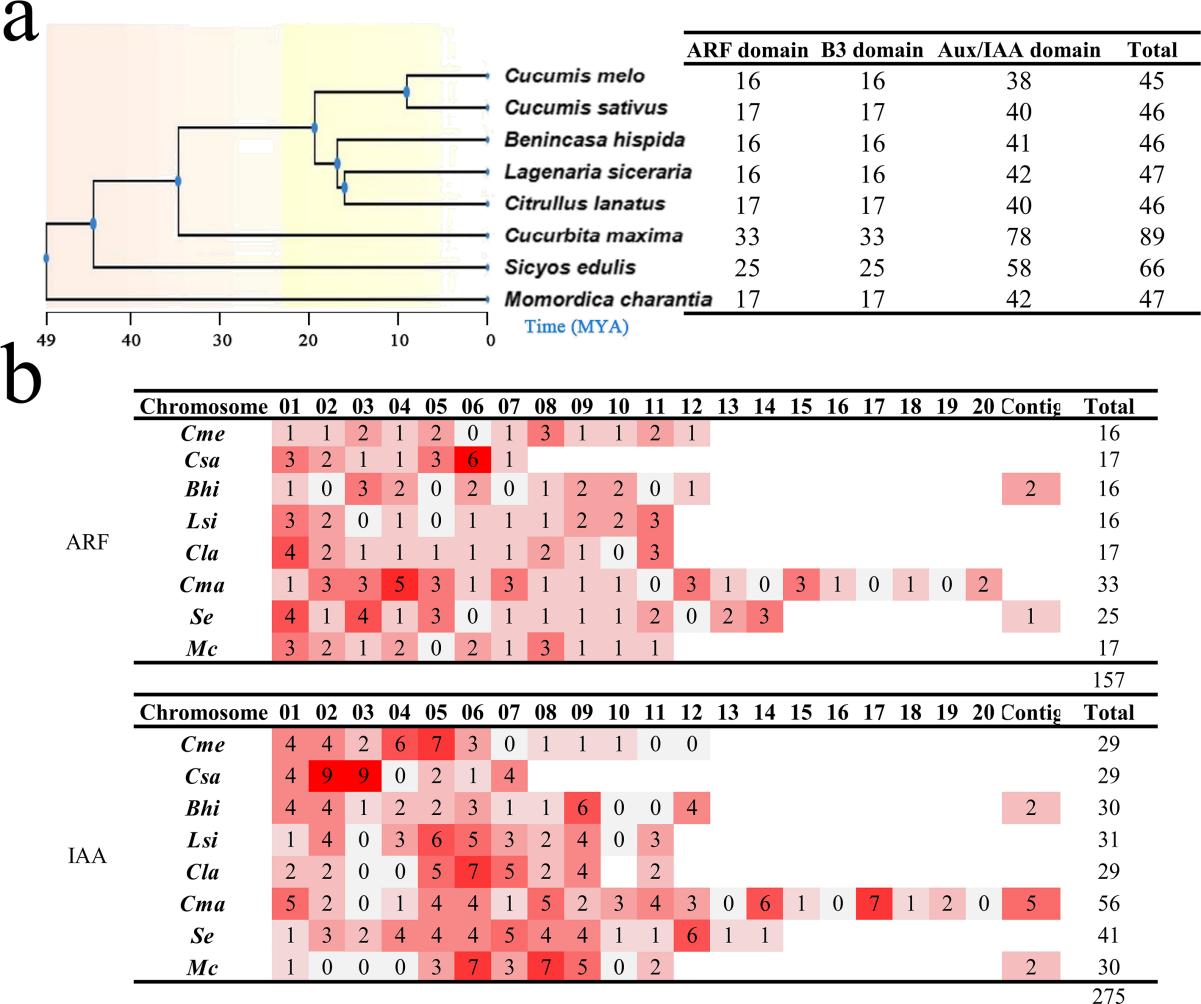
Identification of ARF and IAA genes in eight cucurbit crops. **(a)** Phylogenetic relationships among the eight cucurbit crops (left panel) and the corresponding numbers of ARF and IAA genes identified in each species (right panel). **(b)** Chromosomal distribution patterns of ARF and IAA genes across the eight cucurbit crops. Where numerical labels are present, the highest chromosome number shown represents the total chromosome number offor that species. Genes not assigned to specific chromosomes due to incomplete genome assembly are grouped as “Contig”.

Through comprehensive genome-wide screening (**Figure 1a**), we identified 157 ARF genes distributed across eight cucurbit species as follows: watermelon (17), bottle gourd (16), pumpkin (33), chayote (25), bitter melon (17), wax gourd (16), cucumber (17), and melon (16). Concurrently, we detected 275 IAA genes with the following distribution: watermelon (29), bottle gourd (31), pumpkin (56), chayote (41), bitter melon (30), wax gourd (30), cucumber (29), and melon (29) (**Figure 1b**; **Supplementary Table S1**). The consistent numerical predominance of IAA over ARF genes across all species suggests potential many-to-one interaction patterns between these protein families.

While the size of neither gene family showed a significant correlation with phylogenetic relationships, their combined gene numbers partially reflected evolutionary lineages. Notably, closely related species, melon, cucumber, wax gourd, bottle gourd, and watermelon, exhibited similar total gene counts, contrasting sharply with the phylogenetically distinct pumpkin and chayote. This pattern implies conserved auxin response mechanisms alongside species-specific functional adaptations, with bitter melon representing an exception to this trend.

Chromosomal mapping revealed distinct distribution patterns for both gene families across species. Most genes were localized to specific chromosomal regions, though some remained unmapped. Intriguingly, ARF and IAA genes showed no apparent co-localization within species despite their functional coordination in auxin signaling. In watermelon, for instance, IAA genes clustered predominantly on chromosomes 5, 6, 7, and 9, while ARF genes concentrated on chromosomes 1 and 11. This regional enrichment may reflect targeted selection of agriculturally valuable traits during domestication processes.

### Phylogenetic analysis of ARF and IAA genes in eight cucurbit crops

**Figure 2:**
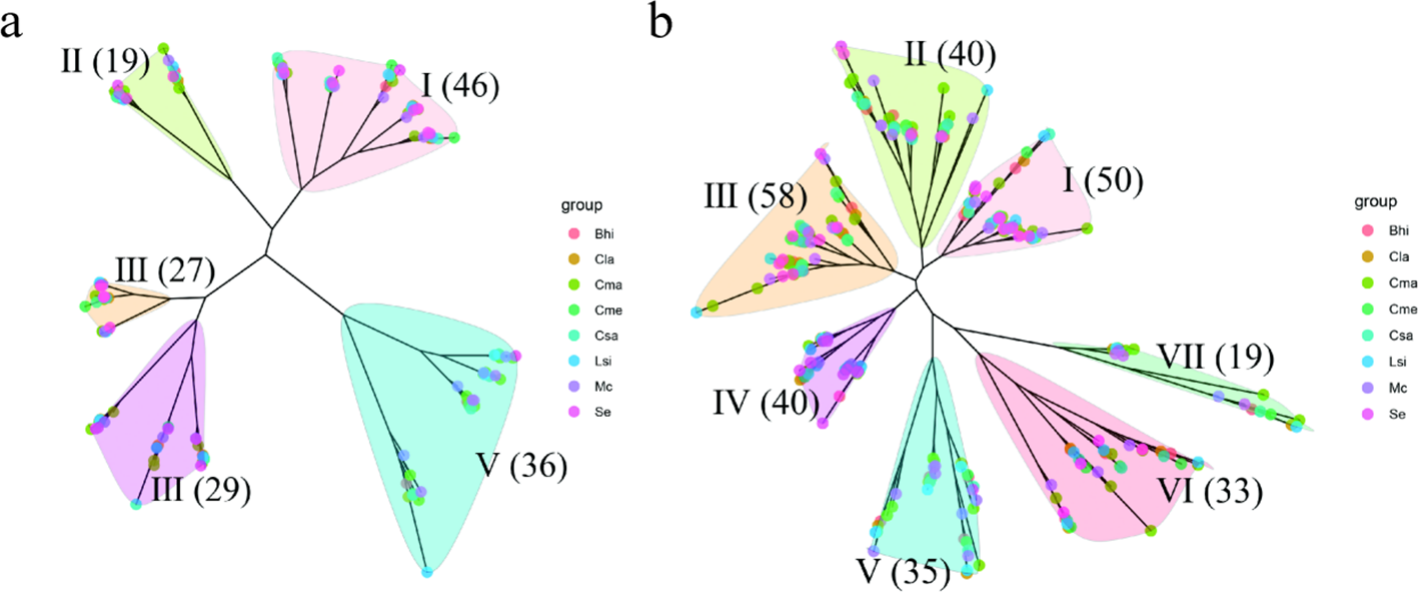
Phylogenetic analysis of ARF and IAA gene families in cucurbits. **(a)** Maximum-likelihood phylogenetic tree of 157 ARF genes constructed using conserved protein domain sequences, revealing five major subfamilies (I–V) indicated by distinct colors. **(b)** Phylogenetic tree of 275 Aux/IAA genes grouped into seven well-supported subfamilies (I–VII), with lineage-specific expansions evident in certain clades.

To elucidate the evolutionary relationships of ARF and IAA genes in cucurbit crops, two unrooted phylogenetic trees were constructed using the Maximum Likelihood (ML) method based on conserved protein domains. The phylogenetic analysis classified 157 ARF genes into 5 distinct subfamilies and 275 IAA genes into 7 well-defined subfamilies (**Figure 2**). Both gene families exhibited clear clustering patterns with multiple evolutionary branches, demonstrating significant functional diversification during evolution. Notably, phylogenetic branches contained orthologous genes from different species, suggesting conserved functions and common ancestral origins.

Comparative analysis of gene structural domains (**Supplementary Figures 2**, **S3**) revealed remarkable conservation of exon-intron organization within phylogenetic clades, whereas pronounced structural variation was observed among different branches. For instance, *SeARF11* and *SeARF14* from *Sechium edule* shared identical gene structures (14 exons and 13 introns) with highly conserved exon-intron distribution patterns. Similar structural conservation was observed among ARF genes, as well as in cross-species comparisons, such as between *CsARF7 (Cucumis sativus*) and *BhiARF1 (Benincasa hispida*). The pervasive conservation of gene structures within phylogenetic clades strongly supports gene duplication as the primary driver of gene family expansion, followed by sub-functionalization during cucurbit evolution.

### Collinearity and natural selection analysis of ARF and IAA genes in eight cucurbit crops

**Figure 3:**
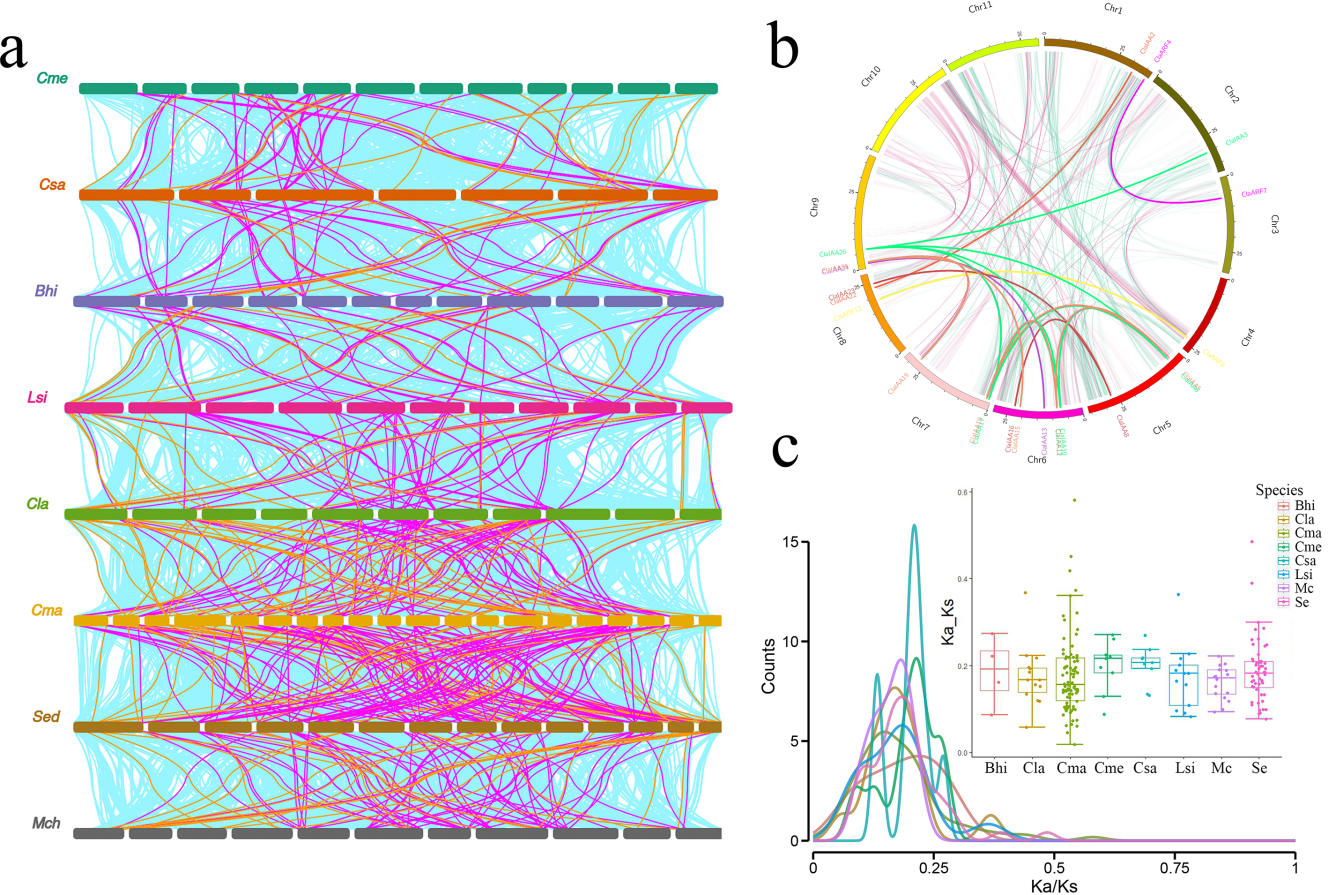
Synteny analysis of ARF and IAA genes in eight cucurbit crops. **(a)** Comparative synteny of ARF (light yellow) and IAA (magenta) orthologous genes across the eight species. Solid lines connect syntenic gene pairs with conserved genomic contexts, demonstrating evolutionary relationships among these auxin signaling components. **(b)** Chromosomal distribution of syntenic ARF and IAA gene pairs in watermelon. The circos plot illustrates both intra-genomic duplications (inner arcs) and inter-chromosomal relationships (connecting lines), highlighting differential expansion patterns between the two gene families. **(c)** Selection pressure analysis of duplicated genes, presented as Ka/Ks ratio distributions for each species. The horizontal dashed line indicates the neutral evolution threshold (Ka/Ks=1), with all values below 0.6 (shaded region) confirming strong purifying selection across both gene families.

To investigate the evolutionary relationships and duplication events of ARF and IAA gene families in cucurbit crops, a comprehensive synteny analysis was performed (**Figure 3a**). The results showed that the ARF gene family contained significantly fewer duplicated genes compared to the IAA family. Numerous highly conserved syntenic blocks were identified across the eight cucurbit species, and most ARF and IAA genes exhibited clear orthologous relationships. These findings indicate that expansion of both gene families primarily resulted from whole-genome duplication (WGD) or segmental duplication (SD) events, while maintaining relatively stable genetic architectures during evolution.

In watermelon, a total of 22 ARF and IAA genes were involved in duplication events, with IAA genes accounting for the majority, suggesting that duplication and functional differentiation of IAA genes may have contributed to environmental adaptation and quality formation during watermelon evolution. The chromosomal distribution of syntenic ARF and IAA gene pairs in watermelon is illustrated in **Figure 3b**. To assess the selection pressure on these duplicated genes, nonsynonymous to synonymous substitutions (Ka/Ks) were calculated (**Figure 3c**). All Ka/Ks values were less than 0.6, demonstrating that both gene families have undergone strong purifying selection in these eight cucurbit crops. Natural selection has maintained the functional stability of encoded proteins by eliminating deleterious nonsynonymous mutations, thereby ensuring trait stability. Furthermore, except for pumpkin (*Cucurbita moschata*) and chayote (*Sechium edule*), the other six species showed relatively small variation in Ka/Ks values, suggesting limited functional divergence of duplicated ARF and IAA genes during their evolutionary history.

### Analysis of cis-acting elements in promoter sequences and gene expression patterns of ARF and IAA genes during fruit development in watermelon and the others cucurbit

**Figure 4:**
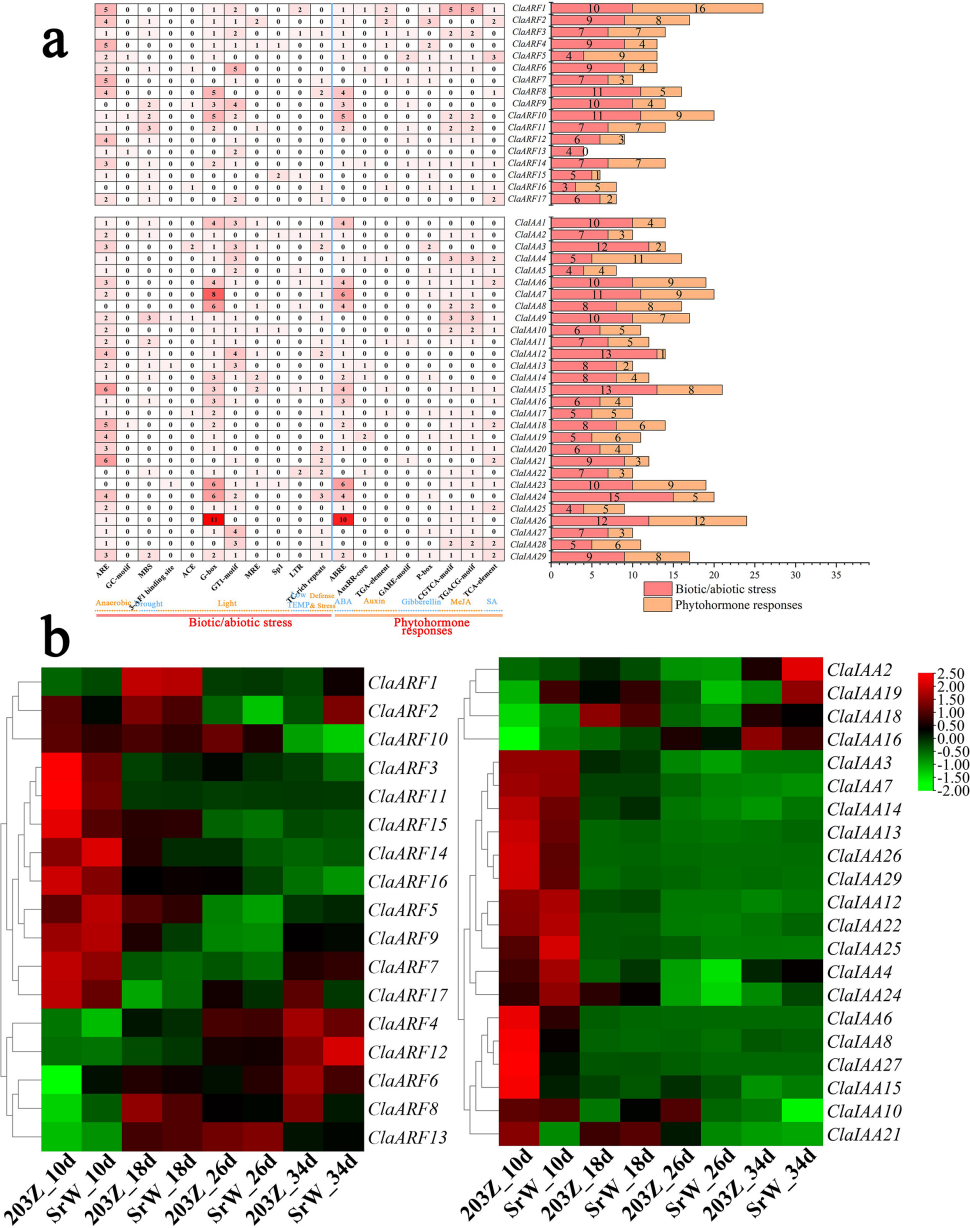
Cis-acting elements in the promoters of ARF and IAA genes and their transcriptional expression patterns during fruit development in watermelon **(a)** Comparative analysis of cis-regulatory elements in the promoter regions (2000 bp upstream of transcription start sites) of ARF and IAA genes from watermelon. **(b)** Expression patterns of ARF and IAA genes during fruit development in different fruits from sweet-predominant (203Z) and acid-predominant (SW) accessions at various days after pollination (DAP).

Building upon previous findings that identified the crucial physiological roles of ARF and IAA genes in cucurbit evolution, we further investigated their transcriptional regulatory mechanisms and potential functions in watermelon. To elucidate the regulatory features of watermelon ARF and IAA genes, cis-acting elements within their promoter regions were systematically analyzed (**Figure 4a**). Interestingly, despite being key regulators of auxin response, these genes showed limited enrichment of growth- and development-related cis-elements in their promoter regions. Therefore, subsequent analyses focused on stress-responsive and hormone-related regulatory elements.

Comparative promoter analysis revealed both conserved and species-specific patterns in the cis-element compositions. Most ARF and IAA genes in watermelon harbored common stress- and hormone-responsive elements, including ARE (antioxidant response element), G-box (light-responsive element), GT1-motif (stress-responsive element), and ABRE (abscisic acid-responsive element), suggesting conserved physiological functions. However, substantial variation was observed in the number and distribution patterns of certain elements among different genes, particularly CGTCA/CGTCG motifs associated with methyl jasmonate (MeJA) responsiveness and TCA elements linked to salicylic acid (SA) signaling. Notably, the high prevalence of ABRE elements, known to enhance stress adaptation and promote fruit ripening, suggests that these genes may function not only in stress responses but also in fruit development and maturation processes, extending their roles beyond classical auxin signaling pathways. Similar regulatory patterns were also detected in phylogenetically distant cucurbit species, indicating evolutionary conservation of these promoter features (**Supplementary figure 4**).

To further assess the functional relevance of ARF and IAA genes during fruit development, published transcriptome datasets were systematically analyzed. After filtering out lowly expressed genes, most ARF and IAA candidates displayed high transcript abundance during early developmental stages, followed by gradual downregulation as fruit maturation advanced (**Figure 4b**). This expression pattern suggests that ARF and IAA genes may play important regulatory roles in the formation of fruit quality traits during the early stages of fruit development in watermelon and other cucurbit crops (**Figure 4b**; **Supplementary figure 6**). Furthermore, transcriptome comparisons among watermelon accessions with contrasting soluble sugar and organic acid contents revealed pronounced stage-dependent expression dynamics. Except for *ClaARF4*, *ClaARF6*, *ClaARF8*, *ClaARF12*, *ClaARF13*, *ClaIAA2*, *ClaIAA18*, and *ClaIAA19*, most of the ARF and IAA genes showed significant upregulation during early fruit development, followed by marked decreases in expression during middle and late developmental stages. These trends were especially evident for IAA genes, indicating a stronger involvement of IAA gene-mediated regulation during early fruit growth.

Previous studies have demonstrated that dynamic changes in soluble sugar and organic acid content begin during early fruit development, indicating that the foundation for final flavor characteristics is established well before ripening (Umer et al., 2020). Accordingly, ARF and IAA genes with pronounced early-stage expression changes represent promising candidates for regulating soluble sugar and organic acid metabolism. These genes may coordinate the expression of key metabolic genes through auxin signaling, thereby influencing the balance between sweetness and acidity, and ultimately shaping watermelon fruit flavor quality. Collectively, the identification of these candidate regulators provides new insights into the molecular mechanisms underlying fruit quality formation in cucurbit crops.

### Exogenous IAA inhibits malate accumulation while promoting hexose biosynthesis

**Figure 5:**
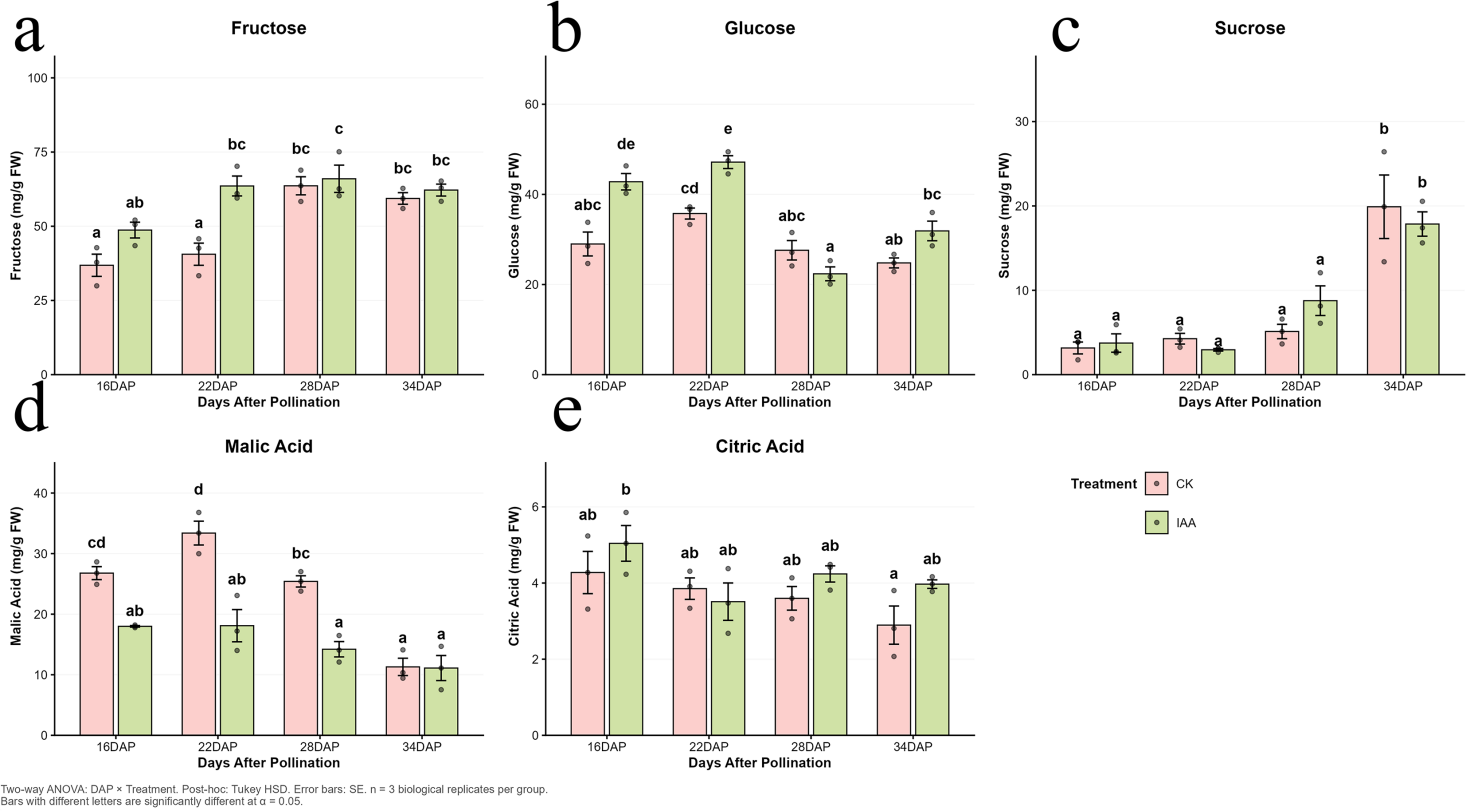
Changes in the contents of fructose **(a)**, glucose **(b)**, sucrose **(c)**, malic acid **(d)**, and citric acid **(e)** at different stages of fruit development under exogenous auxin treatment. Data are presented as mean ± SE (n = 3 biological replicates). Bars with different letters indicate significant differences among treatment × DAP combinations based on Tukey’s HSD test (α = 0.05). Two-way ANOVA was performed to assess the effects of treatment (CK vs. IAA), days after pollination (DAP), and their interaction on metabolite accumulation.

To elucidate the role of auxin in regulating sugar and organic acid metabolism during watermelon fruit development, exogenous indole-3-acetic acid (IAA) was applied, and samples were collected at key developmental stages encompassing fruit set, expansion, and maturation. Quantitative analysis revealed that IAA treatment significantly altered metabolite accumulation during fruit development (**Figure 5**). Fructose and glucose accumulation significantly increased at 16 and 22 DAP (P<0.05), with slight non-significant variation at ripening. In contrast, sucrose content remained largely unchanged throughout fruit development, indicating limited responsiveness to auxin treatment. Among the organic acids, malic acid content significantly decreased at 16, 22, and 28 DAP (P<0.05), whereas no significant difference was observed at maturity. Citric acid content exhibited only minor variation and showed no significant differences between IAA-treated and control fruits across most developmental stages. These results deciphered that auxin promotes hexose accumulation while suppressing malic acid metabolism during the early stages of fruit growth, indicating that exogenous auxin accelerates the early establishment of fruit quality related metabolism in watermelon, thereby promoting the early accumulation of soluble sugars and facilitating a precocious transition toward maturation.

At the molecular level, such auxin-mediated changes in fruit quality are likely associated with the transcriptional regulation of ARF and AUX/IAA genes. As core components of the auxin signaling pathway, ARFs and AUX/IAAs may jointly modulate downstream genes involved in sugar transport, sugar metabolism, and organic acid turnover. Therefore, the observed alterations in metabolite accumulation following IAA treatment may reflect auxin-dependent reprogramming of fruit quality formation through ARF-AUX/IAA-mediated signaling cascades.

### qPCR analysis reveals the involvement of ARF and IAA genes in soluble sugar and organic acid metabolism during watermelon fruit development

**Figure 6:**
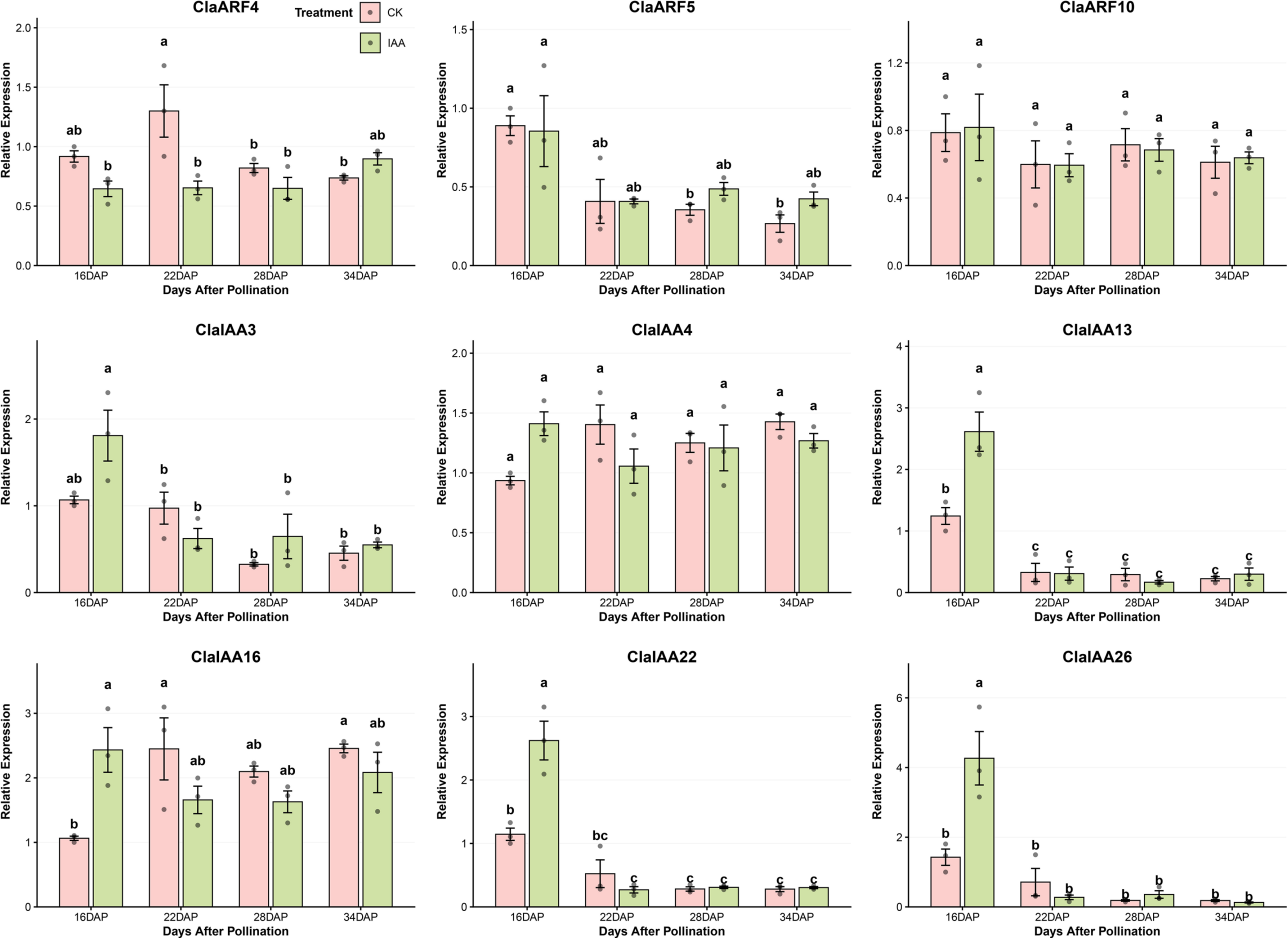
Expression profiles of auxin-responsive genes during watermelon fruit development under exogenous auxin treatment. Relative expression levels of three *ARF* genes (*ClaARF4*, *ClaARF5*, *ClaARF10*) and six *Aux/IAA* genes (*ClaIAA3*, *ClaIAA4*, *ClaIAA13*, *ClaIAA16*, *ClaIAA22*, *ClaIAA26*) were quantified by RT-qPCR at 16, 22, 28, and 34 DAP in control (CK) and IAA-treated fruits. Data are presented as mean ± standard error (SE) from three biological replicates, each analyzed in triplicate as technical replicates. Expression levels were normalized to the reference gene *ClaACTIN*.

To identify potential ARF and IAA candidate genes involved in regulating sugar-acid balance during early watermelon fruit development, nine genes exhibiting significant expression differences in early developmental stages were selected for qPCR validation (**Figure 6**). These candidate genes included watermelon orthologs of tomato *SlARF4* and *SlARF6 (ClaARF5* and *ClaARF4*, respectively), which have been functionally characterized in tomato sucrose biosynthesis (Sagar et al., 2013; Yuan et al., 2019), along with seven additional genes (*ClaIAA13*, *ClaIAA22*, *ClaIAA26*, etc.) showing high absolute expression levels during early fruit development.

Comparative analysis of expression patterns under exogenous IAA treatment revealed that *ClaIAA13*, *ClaIAA22*, and *ClaIAA26* exhibited significantly higher transcript abundance during early fruit development compared to untreated controls (CK), with expression profiles closely matching the transcriptome data. These findings strongly suggest that these three IAA genes may mediate auxin signaling to coordinate the expression of downstream targets involved in sugar and organic acid metabolism, thereby influencing the dynamic balance of these key flavor determinants during early fruit development. The consistent validation of both transcriptomic and qPCR data strengthens the reliability of these candidates as key regulators of fruit quality formation in watermelon.

### Functional differentiation of *ClaIAA22* during CA to CM acclimation and the early stage of watermelon fruit development

**Figure 7:**
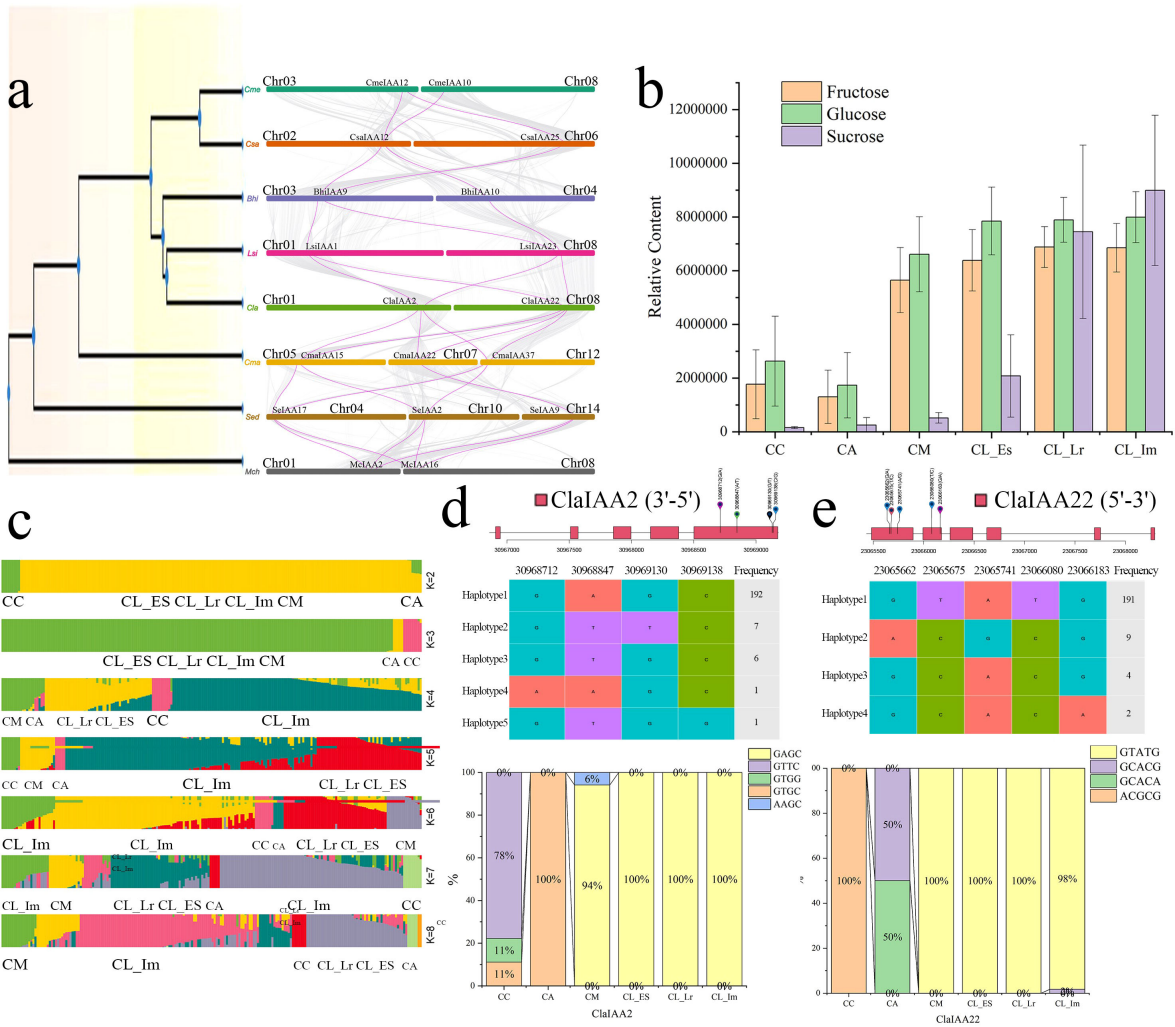
Domestication analysis of soluble sugars and identification of differentially selected genes in watermelon. **(a)** Evolutionary relationships of *ClaIAA22* and *ClaIAA2* among different Cucurbitaceae species. **(b)** Dynamic changes in glucose, fructose, and sucrose content across different domestication stages of watermelon. **(c)** Population structure of 211 watermelon accessions at optimal clustering (K=4). **(d)** SNP variations in *ClaIAA2* during domestication. **(e)** SNP variations in *ClaIAA22* during domestication.

Sweetness, a key domestication trait in watermelon, is primarily determined by soluble sugar content. Building upon our previously published data (Yuan et al., 2023), the materials were classified into six distinct populations: *C. colocynthis* (CC), *C. amarus* (CA), *C. mucosospermus* (CM), *C. lanatus* edible seed (CL_Es), *C. lanatus* landrace (CL_Lr), and *C. lanatus* improved (CL_Im). Population structure analysis of natural watermelon accessions using the STRUCTURE software (K = 4) clearly differentiated four distinct groups: (1) CC as an independent lineage; (2) CA and CM forming a closely related cluster; (3) landraces sharing partial genetic background with seed-type watermelons; and (4) improved cultivars showing genetic admixture with landraces (**Figure 7c**). This analysis reveals the close genetic relationships between cultivated and seed-type watermelons, with clear domestication boundaries yet evident genetic introgression between improved cultivars and landraces.

Metabolomic profiling of sucrose, glucose, and fructose content across different domestication groups revealed significant metabolic shifts during watermelon evolution. All three soluble sugars were relatively low in early domestication stages but increased markedly at specific transitions (**Figure 7b**). Notably, glucose and fructose content rose dramatically during the *C. amarus* (CA) to *C. mucosospermus* (CM) transition, while sucrose accumulation showed a pronounced increase later in domestication, specifically during the shift from edible-seed types (CL_Es) to landraces (CL_Lr). To identify potential genetic regulators underlying these metabolic changes, six highly expressed ARF and IAA genes were analyzed for SNP genotyping and allele frequency (**Figures 7d, e**; **Supplementary figure 6**). Four genes, *ClaARF4*, *ClaARF15*, *ClaIAA4*, and *ClaIAA22*, that underwent significant allelic differentiation during the CA-to-CM transition and remained stably fixed in subsequent domestication stages. Site-specific allelic effect analysis revealed that multiple SNP variants within *ClaIAA22* were significantly associated with soluble sugar accumulation, with SNPs at positions Chr08:23065662 and Chr08:23065741 exhibiting particularly pronounced effects (**Supplementary Figure 7b**). Considering the qPCR results of each gene (we propose that *ClaIAA22* may be a key gene regulating the sharp increase in glucose and fructose contents during the early stages of watermelon fruit development. When mapped across the genomes of different cucurbit species, we found that *ClaIAA22* appeared to have co-evolved with *ClaIAA22* (**Figure 7a**). Interestingly, the genotypic variations of *ClaIAA2* during domestication exhibited a similar accumulation trend of glucose and fructose (**Figure 7d**; **Supplementary Table S3**). However, its expression pattern during fruit development showed no significant association with sugar and acid metabolism, possibly due to differences in the promoter regions (**Figure 4**).

## Discussion

Auxin plays a pivotal role in regulating plant growth, development, and stress responses (Du et al., 2020; Singh et al., 2021; Tiwari et al., 2023). The ARF and IAA gene families involved in auxin signaling have been individually investigated in several cucurbit crops, including cucumber (Wu et al., 2014), melon (Wu et al., 2020), and watermelon (Hu et al., 2023; Anees et al., 2024), with studies focusing on single species or individual gene families that have primarily emphasized fruit growth, ripening, or stress responses. On the other hand, the modulation ARF and IAA genes across the Cucurbitaceae and their relevance to fruit quality traits, particularly soluble sugar and organic acid metabolism during domestication and fruit development, has been limited. This study presents the first integrated, family-wide comparative analysis of ARF and IAA genes across eight cucurbit crops, linking their evolutionary and regulatory features, with particular emphasis on sugar and organic acid metabolism, the principal fruit flavor components, and domestication-driven differentiation in watermelon (Ren et al., 2018; Martí et al., 2019). Furthermore, through a combination of SNP variation screening of candidate genes, we identified critical periods of sugar content differentiation and potential candidate genes involved in this process.

Comparative genomics shows that most genes cluster into well-supported orthologous groups shared across species, reflecting both the deep conservation of core functions and lineage-specific gains, losses, and innovations (Gabaldón and Koonin, 2013). In this study, comparative genomic analysis revealed varying numbers of ARF and IAA gene family members that were highly conserved across the Cucurbitaceae, with most genes forming well-supported orthologous groups shared among species. As each phylogenetic clade contained members from all eight species, the diversification of both gene families predates species divergence, indicating that their core regulatory functions were established early in cucurbit evolution; this observation is consistent with previous studies (Remington et al., 2004). Concomitantly, Ka/Ks values of less than 0.6 demonstrated that most duplicated ARF and IAA genes experienced strong purifying selection, highlighting a certain degree of evolutionary conservation in protein function and signaling integrity within each species (Hurst, 2002). Furthermore, promoter analysis of ARF and IAA genes across watermelon, melon, and cucumber revealed several conserved cis-elements, including ABRE, which is associated with hormone-mediated ripening hormone during fruit development. This ripening is a developmental phase involving numerous physiological and biochemical processes (Velasquez et al., 2016; Alabdallah et al., 2017; Matthes et al., 2019; McLaughlin et al., 2021). Based on these evidences, this study hypothesized the expression of ARF and IAA genes act as key regulator of watermelon fruit development. Interestingly, focusing on the patterns of soluble sugars and organic acids, which vary early during fruit development (Ren et al., 2020; Gao et al., 2023), transcriptomes analysis sweet- and sour-fleshed varieties, revealed that the expression of most ARF and IAA genes differed markedly during early fruit development but remained stable at later stages. Similar regulatory roles for ARF and IAA genes have also been reported in crops such as maize, citrus, and mango (Xie et al., 2018; Zhou et al., 2023; Wang et al., 2024). These findings suggest that ARF and IAA genes may play foundational roles in establishing these metabolic changes during early fruit development.

ARF and IAA genes are known to respond to auxin and regulate downstream gene expression (Jin et al., 2024; Kubalová et al., 2024). Inadequate auxin signaling associated with lower sugar and auxin contents, and higher acid levels in watermelon fruit (Wang et al., 2021). In this study, exogenous auxin application in watermelon increased glucose and fructose levels during early fruit development while reducing malic acid content. In parallel, ClaIAA13, ClaIAA22, and ClaIAA26 exhibited auxin-responsive expression patterns consistent with the observed metabolite change (**Figure 6**). These genes included both highly expressed genes and homologs previously reported to have regulatory roles in other crops (Sagar et al., 2013; Yuan et al., 2019). Similarly, the application of exogenous IAA has been shown to up-regulate 19 citrus *ARF* genes in sweet orange (Li et al., 2015). The levels of glucose, fructose, and sucrose have been significantly higher in tomato fruits overexpressing the *SlARF6A* gene than in wild-type fruits, particularly at the orange and red stages of fruit development. The expression changes of these candidate genes imply that they may regulate the conversion between soluble sugars and organic acids by modulating enzymes such as hexokinase (HK) and phosphofructokinase (PEK), using glucose as an intermediary. In’Royal Gala’ apple, these enzymes have been reported to be upregulated by exogenous auxin application and identified as major contributors to sink strength (Jing et al., 2022). Mechanistically, the application of exogenous auxin has been reported to increase auxin signaling, thereby enhancing sink strength through the upregulation of polygalacturonase, β-amylase, and sugar transporter enzymes, which may increase sink size and activity, whereas the application of auxin inhibitors has been associated with increased acid accumulation in plants (Balasubramanian et al., 2024). Through such regulation, ARF and IAA genes may mediate the metabolic transition from organic acid accumulation to soluble sugar enrichment during fruit development and ripening (Hou et al., 2020).

Domestication analysis provides important insights into the phenotypic and genetic variations shaped by past human selection for agronomic traits (Jayakodi et al., 2019). Analyzing previously published datasets, we figured out that glucose and fructose underwent strong selection from the wild ancestor group (CA) to the cultivated landrace group (CM), while sucrose accumulation increased from early to late landraces (CL_Es to CL_Lr) during watermelon fruit domestication. This pattern suggests that glucose and fructose were the first sugars selected during watermelon domestication, contributing to the initial improvement in sweetness following the emergence of edible-seeded watermelons. Further enhancement and stabilization of total sugar content likely occurred with the development of local landraces. Thus, the shift in watermelon fruit flavor from bitter in wild types to sweet in modern cultivars was primarily driven by continuous selection for sugar content and the simultaneous elimination of bitterness-related traits during domestication (Guo et al., 2019; Jayakodi et al., 2019). Furthermore, genotype classification of SNPs in highly expressed genes revealed that *ClaARF4*, *ClaARF15*, *ClaIAA4*, and *ClaIAA22* mirrored the patterns of glucose and fructose patterns, suggesting regulatory roles. Both horizontal (fruit developmental stages) and vertical (domestication trajectory) analyses consistently pointed to *ClaIAA22* as a positive regulator of glucose and fructose biosynthesis. Taken together, our findings strongly suggest that *ClaIAA22* underwent functional differentiation during the domestication transition from CA to CM and may serve as a strong candidate domestication-related regulator in early fruit development by modulating glucose and fructose synthesis. Importantly, this regulatory function appears to have been maintained throughout the domestication process and continues to influence sugar accumulation in modern watermelon cultivars. Although the regulatory role of this gene is exerted at an early stage, we consider it to be meaningful for fruit development and production. At the molecular level, these auxin-mediated changes in fruit quality may be attributed to the transcriptional regulation of ARF and AUX/IAA genes. As core components of the auxin signaling pathway, ARFs and AUX/IAAs likely coordinately regulate downstream genes involved in sugar transport, sugar metabolism, and organic acid turnover. Thus, the alterations in metabolite accumulation observed following IAA treatment may reflect an auxin-dependent reprogramming of fruit quality formation via ARF-AUX/IAA-mediated signaling cascades.

## Conclusion

This study provides the first integrated evidence that ARF and IAA gene families coordinate sugar and organic acid metabolism in Cucurbitaceae through evolutionarily conserved yet functionally divergent mechanisms. Genome-wide analyses identified 157 ARF and 275 IAA genes across eight cucurbit species, with purifying selection (Ka/Ks < 0.6) maintaining functional stability. Promoter regions were enriched with ABA-responsive elements that are involved in fruit development, and transcriptome profiling revealed peak expression during early fruit development, coinciding with metabolic shifts. Exogenous auxin treatment increased glucose and fructose by 23-35% while reducing malate by 18%, with *ClaIAA13*, *ClaIAA22*, and *ClaIAA26* identified as key auxin-responsive regulators. Domestication analysis showed strong selection for hexose accumulation during the transition from wild types CA to cultivated types CM, contributing to the sweet taste of modern watermelon cultivars. Notably, *ClaIAA22* emerged as a key candidate gene whose expression dynamics concomitantly paralleled the trend of hexose increase and malate reduction during both watermelon fruit development and domestication. This gene underwent functional differentiation during domestication while maintaining regulatory activity in early-stage hexose biosynthesis, representing a promising target for molecular breeding to enhance fruit sweetness in watermelon and related crops. Future directions include functional validation of *ClaIAA22* and other auxin-responsive regulators through gene editing approaches, as well as the translation of these findings into breeding applications via marker-assisted selection or gene pyramiding for enhanced fruit sweetness in cucurbits.

## Data Availability

The original contributions presented in the study are included in the article/**Supplementary Material**. Further inquiries can be directed to the corresponding authors.

## References

[B1] Alabdallah O. Ahou A. Mancuso N. Pompili V. Macone A. Pashkoulov D. . (2017). The arabidopsis polyamine oxidase/dehydrogenase 5 interferes with cytokinin and auxin signaling pathways to control xylem differentiation. J. Exp. Bot. 68, 997–1012. doi: 10.1093/jxb/erw510 28199662

[B2] Anees M. Gao L. Gong C. Umer M. J. Yuan P. Hongju Z. . (2023). AUX/IAA gene Cla004102, is involved in synergistic regulation of various endogenous hormones, regulating flesh firmness in watermelon. Sci. Hortic. 310, 111719. doi: 10.1016/j.scienta.2022.111719 38826717

[B3] Anees M. Zhu H. Umer M. J. Gong C. Yuan P. Xuqiang L. . (2024). Identification fication of an AUX/IAA regulator for flesh firmness using combined gwas and bulked segregant rna-seq analysis in watermelon. Hortic. Plant J. 10, 1198–1213. doi: 10.1016/j.hpj.2023.05.018 38826717

[B4] Balasubramanian V. K. Rivas-Ubach A. Winkler T. Mitchell H. Moran J. Ahkami A. H. . (2024). Modulation of polar auxin transport identifies the molecular determinants of source-sink carbon relationships and sink strength in poplar. Tree Physiol. 44, 82–101. doi: 10.1093/treephys/tpad073 37265358 PMC11898627

[B5] Benjamini Y. Hochberg Y. (1995). Controlling the False Discovery Rate: A Practical and Powerful Approach to Multiple Testing. Journal of the Royal Statistical Society. Series B (Methodological), 57(1), 289–300. Available online at: http://www.jstor.org/stable/2346101 37265358

[B6] Bu H. Sun X. Yue P. Qiao J. Sun J. Wang A. . (2022). The MdAUX/IAA2 transcription repressor regulates cell and fruit size in apple fruit. Int. J. Mol. Sci. 23. doi: 10.3390/ijms23169454 36012719 PMC9408813

[B7] Die J. V. Gil J. Millan T. (2018). Genome-wide identification of the auxin response factor gene family in cicer arietinum. BMC Genomics 19, 301. doi: 10.1186/s12864-018-4695-9 29703137 PMC5921756

[B8] Du M. Spalding E. P. Gray W. M. (2020). Rapid auxin-mediated cell expansion. Annu. Rev. Plant Biol. 71, 379–402. doi: 10.1146/annurev-arplant-073019-025907 32131604 PMC7733314

[B9] Durán-Soria S. Pott D. M. Osorio S. Vallarino J. G. (2020). Sugar signaling during fruit ripening. Front. Plant Sci. 11, 564917. doi: 10.3389/fpls.2020.564917 32983216 PMC7485278

[B10] Gabaldón T. Koonin E. V. (2013). Functional and evolutionary implications of gene orthology. Nat. Rev. Genet. 14, 360–366. doi: 10.1038/nrg3456 23552219 PMC5877793

[B11] Gan D. Zhuang D. Ding F. Yu Z. Zhao Y. (2013). Identification and expression analysis of primary auxin-responsive AUX/IAA gene family in cucumber (Cucumis sativus). J. Genet. 92, 513–521. doi: 10.1007/s12041-013-0306-3 24371172

[B12] Gao L. Zhao S. Lu X. He N. Zhu H. Junling D. . (2018). Comparative transcriptome analysis reveals key genes potentially related to soluble sugar and organic acid accumulation in watermelon. PloS One 13, e190096. doi: 10.1371/journal.pone.0190096 29324867 PMC5764247

[B13] Gao W. She F. Sun Y. Han B. Wang X. Gang X. . (2023). Transcriptome analysis reveals the genes related to water-melon fruit expansion under low-light stress. Plants 12, 935. doi: 10.3390/plants12040935 36840282 PMC9958833

[B14] Guo S. Zhao S. Sun H. Wang X. Wu S. Lin T. . (2019). Resequencing of 414 cultivated and wild watermelon accessions identifies selection for fruit quality traits. Nat. Genet. 51, 1616–1623. doi: 10.1038/s41588-019-0518-4 31676863

[B15] Hammes U. Z. Pedersen B. P. (2024). Structure and function of auxin transporters. Annu. Rev. Plant Biol. 75, 185–209. doi: 10.1146/annurev-arplant-070523-034109 38211951

[B16] Han L. Li M. Li C. Zhao B. Wang Z. Yu L. . (2026). Arf3-mediated auxin signaling is essential for sex determination in cucumber. Sci. (New York) 391, 59–63. doi: 10.1126/science.adv2006 41379936

[B17] He W. Yang J. Jing Y. Xu L. Yu K. Xiaodong F. (2023). Ngenomesyn : an easy-to-use and flexible tool for publication-ready visualization of syntenic relationships across multiple genomes. Bioinformatics 39. doi: 10.1093/bioinformatics/btad121 36883694 PMC10027429

[B18] He X. Zheng Y. Yang S. Wang Y. Lin Y. Biao J. . (2024). Combined genomic, transcriptomic, and metabolomic analyses provide insights into the fruit development of bottle gourd (lagenaria siceraria). Hortic. Res. 12, uhae335. doi: 10.1093/hr/uhae335 40051576 PMC11883228

[B19] Hou Y. Li H. Zhai L. Xie X. Li X. Biao S. (2020). Identification and functional characterization of the AUX/IAA gene VcIAA27 in blueberry. Plant Signal. Behav. 15, 1700327. doi: 10.1080/15592324.2019.1700327 31822153 PMC7012069

[B20] Hu Q. Yang J. Meng L. Liu J. Tian S. (2023). Genome-wide identification of AUX/IAA genes in watermelon reveals a crucial role for ClIAA16 during fruit ripening. Horticulturae 9. doi: 10.3390/horticulturae9111167 30654563

[B21] Hurst L. D. (2002). The ka/ks ratio: diagnosing the form of sequence evolution. Trends Genet. 18, 486. doi: 10.1016/s0168-9525(02)02722-1 12175810

[B22] Jayakodi M. Schreiber M. Mascher M. (2019). Sweet genes in melon and watermelon. Nat. Genet. 51, 1572–1573. doi: 10.1038/s41588-019-0529-1 31676862

[B23] Jin F. Zhu L. Hou L. Li H. Li L. Guanghui X. . (2024). Auxin resistant 2 and short hypocotyl 2 regulate cotton fiber initiation and elongation. Plant Physiol. 195, 2032–2052. doi: 10.1093/plphys/kiae183 38527791

[B24] Jing S. U. Wei-fang C. Ling-cheng Z. Bai-yun L. I. Feng-wang M. A. Mingjun L. . (2022). Response of carbohydrate metabolism-mediated sink strength to auxin in shoot tips of apple plants. J. Integr. Agric. 21, 422–433. doi: 10.1016/S2095-3119(20)63593-6

[B25] Kenea F. T. He N. Lu X. Luo X. Zhu H. Wenge L. (2025). Watermelon fruit metabolome gene discovery and its application in breeding: a review. Front. Plant Sci. 16, 1687406. doi: 10.3389/fpls.2025.1687406 41190210 PMC12581998

[B26] Kubalová M. Müller K. Dobrev P. I. Rizza A. Jones A. M. Fendrych M. (2024). Auxin co-receptor IAA17/AXR3 controls cell elongation in arabidopsis thaliana root solely by modulation of nuclear auxin pathway. New Phytol. 241, 2448–2463. doi: 10.1111/nph.19557 38308183

[B27] Kumar S. Stecher G. Tamura K. (2016). Mega7 : molecular evolutionary genetics analysis version 7.0 for bigger datasets. Mol. Biol. Evol. 33, 1870–1874. doi: 10.1093/molbev/msw054 27004904 PMC8210823

[B28] Li Y. Han S. Qi Y. (2023). Advances in structure and function of auxin response factor in plants. J. Integr. Plant Biol. 65, 617–632. doi: 10.1111/jipb.13392 36263892

[B29] Li S. OuYang W. Hou X. Xie L. Hu C. Jinzhi Z. (2015). Genome-wide identification, isolation and expression analysis of auxin response factor (arf) gene family in sweet orange (citrus sinensis). Front. Plant Sci. 6, 119. doi: 10.3389/fpls.2015.00119 25870601 PMC4378189

[B30] Livak K. J. Schmittgen T. D. (2001). Analysis of relative gene expression data using real-time quantitative pcr and the 2–δδct method. Methods 25, 402–408. doi: 10.1006/meth.2001.1262 11846609

[B31] Martí R. Sánchez G. Valcárcel M. Roselló S. Cebolla-Cornejo J. (2019). High throughput ft-mir indirect analysis of sugars and acids in watermelon. Food Chem. 300, 125227. doi: 10.1016/j.foodchem.2019.125227 31351262

[B32] Matthes M. S. Best N. B. Robil J. M. Malcomber S. Gallavotti A. McSteen P. (2019). Auxin evodevo: conservation and diversification of genes regulating auxin biosynthesis, transport, and signaling. Mol. Plant 12, 298–320. doi: 10.1016/j.molp.2018.12.012 30590136

[B33] McLaughlin H. M. Ang A. Ostergaard L. (2021). Noncanonical auxin signaling. Cold Spring Harbor Perspect. Biol. 13. doi: 10.1101/cshperspect.a039917 33431583 PMC8091950

[B34] Moeen-Ud-Din M. Yang S. Wang J. (2023). Auxin homeostasis in plant responses to heavy metal stress. Plant Physiol. Biochem. 205, 108210. doi: 10.1016/j.plaphy.2023.108210 38006792

[B35] Nie J. Huang H. Wu S. Lin T. Zhang L. Lijun L. . (2025). Molecular regulation and domestication of parthenocarpy in cucumber. Nat. Plants 11, 176–190. doi: 10.1038/s41477-024-01899-2 39814959

[B36] Powers S. K. Holehouse A. S. Korasick D. A. Schreiber K. H. Clark N. M. Hongwei J. . (2019). Nucleo-cytoplasmic partitioning of arf proteins controls auxin responses in arabidopsis thaliana. Mol. Cell 76, 177–190. doi: 10.1016/j.molcel.2019.06.044 31421981 PMC6778021

[B37] Pritchard J. K. Stephens M. Donnelly P. (2000). Inference of population structure using multilocus genotype data. Genetics 155, 945–959. doi: 10.1093/genetics/155.2.945 10835412 PMC1461096

[B38] Remington D. L. Vision T. J. Guilfoyle T. J. Reed J. W. (2004). Contrasting modes of diversification in the AUX/IAA and arf gene families. Plant Physiol. 135, 1738–1752. doi: 10.1104/pp.104.039669 15247399 PMC519086

[B39] Ren Y. Guo S. Zhang J. He H. Sun H. Shouwei T. . (2018). A tonoplast sugar transporter underlies a sugar accumulation qtl in watermelon. Plant Physiol. 176, 836–850. doi: 10.1104/pp.17.01290 29118248 PMC5761790

[B40] Ren Y. Sun H. Zong M. Guo S. Ren Z. Jianyu Z. . (2020). Localization shift of a sugar transporter contributes to phloem unloading in sweet watermelons. New Phytol. 227, 1858–1871. doi: 10.1111/nph.16659 32453446

[B41] Roth C. Liberles D. A. (2006). A systematic search for positive selection in higher plants (embryophytes). BMC Plant Biol. 6, 12. doi: 10.1186/1471-2229-6-12 16784532 PMC1540423

[B42] Sagar M. Chervin C. Mila I. Hao Y. Roustan J. P. Benichou M. . (2013). Slarf4, an auxin response factor involved in the control of sugar metabolism during tomato fruit development. Plant Physiol. 161, 1362–1374. doi: 10.1104/pp.113.213843 23341361 PMC3585602

[B43] Saurabh S. Prasad D. Masi A. Vidyarthi A. S. (2022). Next generation sequencing and transcriptome analysis for identification of arf and AUX/IAA in pointed gourd (trichosanthes dioica roxb.), A non-model plant. Sci. Hortic. 301, 111152. doi: 10.1016/j.scienta.2022.111152 38826717

[B44] Singh H. Bhat J. A. Singh V. P. Corpas F. J. Yadav S. R. (2021). Auxin metabolic network regulates the plant response to metalloids stress. J. Hazard. Mater. 405, 124250. doi: 10.1016/j.jhazmat.2020.124250 33109410

[B45] Tiwari M. Kumar R. Subramanian S. Doherty C. J. Jagadish S. V. K. (2023). Auxin–cytokinin interplay shapes root functionality under low-temperature stress. Trends Plant Sci. 28, 447–459. doi: 10.1016/j.tplants.2022.12.004 36599768

[B46] Umer M. J. Bin S. L. Gebremeskel H. Zhao S. Yuan P. Hongju Z. . (2020). Identification of key gene networks controlling organic acid and sugar metabolism during watermelon fruit development by integrating metabolic phenotypes and gene expression profiles. Hortic. Res. 7, 193. doi: 10.1038/s41438-020-00416-8 33328462 PMC7705761

[B47] Untergasser A. Cutcutache I. Koressaar T. Ye J. Faircloth B. C. Rozen S.G. . (2012). Primer3--new capabilities and interfaces. Nucleic Acids Res. 40, e115. doi: 10.1093/nar/gks596 22730293 PMC3424584

[B48] Velasquez S. M. Barbez E. Kleine-Vehn J. Estevez J. M. (2016). Auxin and cellular elongation. Plant Physiol. 170, 1206–1215. doi: 10.1104/pp.15.01863 26787325 PMC4775141

[B49] Wang M. Feng G. Yang Z. Wu J. Liu B. Xiaoheng X. . (2023). Genome-wide characterization of the AUX/IAA gene family in orchardgrass and a functional analysis of DgIAA21 in responding to drought stress. Int. J. Mol. Sci. 24. doi: 10.3390/ijms242216184 38003372 PMC10671735

[B50] Wang H. Huang Y. Li Y. Cui Y. Xiang X. Yidong Z. . (2024). An arf gene mutation creates flint kernel architecture in dent maize. Nat. Commun. 15, 2565. doi: 10.1038/s41467-024-46955-9 38519520 PMC10960022

[B51] Wang Y. Tang H. DeBarry J. D. Tan X. Li J. Wang X. . (2012). Mcscanx : a toolkit for detection and evolutionary analysis of gene synteny and collinearity. Nucleic Acids Res. 40. doi: 10.1093/nar/gkr1293 22217600 PMC3326336

[B52] Wang J. Wang Y. Zhang J. Ren Y. Li M. Yongtao Y. . (2021). The nac transcription factor clnac68 positively regulates sugar content and seed development in watermelon by repressing clinv and clgh3.6. Hortic. Res. 8, 214. doi: 10.1038/s41438-021-00649-1 34593776 PMC8484586

[B53] Wang D. Zhang Y. Zhang Z. Zhu J. Yu J. (2010). Kaks_calculator 2.0: a toolkit incorporating gamma-series methods and sliding window strategies. Genom. Proteomics Bioinf. 8, 77–80. doi: 10.1016/S1672-0229(10)60008-3 20451164 PMC5054116

[B54] Weijers D. Benkova E. Jager K. E. Schlereth A. Hamann T. Kientz M. . (2005). Developmental specificity of auxin response by pairs of arf and AUX/IAA transcriptional regulators. EMBO J. 24, 1874–1885. doi: 10.1038/sj.emboj.7600659 15889151 PMC1142592

[B55] Wu J. Liu S. Guan X. Chen L. He Y. Jie W. . (2014). Genome-wide identification and transcriptional profiling analysis of auxin response-related gene families in cucumber. BMC Res. Notes 7, 218. doi: 10.1186/1756-0500-7-218 24708619 PMC4108051

[B56] Wu B. Wang L. Pan G. Li T. Li X. Jinghong H. . (2020). Genome-wide characterization and expression analysis of the auxin response factor (arf) gene family during melon (cucumis melo l.) fruit development. Protoplasma 257, 979–992. doi: 10.1007/s00709-020-01484-2 32043172 PMC7203594

[B57] Xie R. Ge T. Zhang J. Pan X. Ma Y. Shilai Y. . (2018). The molecular events of IAA inhibiting citrus fruitlet abscission revealed by digital gene expression profiling. Plant Physiol. Biochem. 130, 192–204. doi: 10.1016/j.plaphy.2018.07.006 29990772

[B58] Xing Y. Zhang X. Feng Z. Ni W. Xie H. Yafei G. . (2024). Optimizing 'red fuji' apple quality: auxin-mediated calcium distribution via fruit-stalk in bagging practices. Food Chem. 463, 141126. doi: 10.1016/j.foodchem.2024.141126 39276559

[B59] Yang E. Yang H. Li C. Zheng M. Song H. Zou X. . (2022). Genome-wide identification and expression analysis of the AUX/IAA gene family of the drumstick tree (moringa oleifera lam.) reveals regulatory effects on shoot regeneration. Int. J. Mol. Sci. 23. doi: 10.3390/ijms232415729 36555370 PMC9779525

[B60] Yuan Y. Xu X. Gong Z. Tang Y. Wu M. Fang Y. . (2019). Auxin response factor 6a regulates photosynthesis, sugar accumulation, and fruit development in tomato. Hortic. Res. 6, 85. doi: 10.1038/s41438-019-0167-x 31645946 PMC6804849

[B61] Yuan P. Xu C. He N. Lu X. Zhang X. Jianli S. . (2023). Watermelon domestication was shaped by stepwise selection and regulation of the metabolome. Sci. China Life Sci. 66, 579–594. doi: 10.1007/s11427-022-2198-5 36346547

[B62] Zhang R. Jia G. Diao X. (2023). Genehapr: an r package for gene haplotypic statistics and visualization. BMC Bioinf. 24, 199. doi: 10.1186/s12859-023-05318-9 37189023 PMC10186671

[B63] Zhang Q. Wang K. Yang B. Guo X. Zhao L. Chunhua C. . (2025). Identification of auxin response factor in the pan-genome and their expression pattern analysis in cucumber. AIMS Mol. Sci. 12, 318–340. doi: 10.3934/molsci.2025019

[B64] Zhang M. Xue Y. Xu S. Jin X. Man X. (2024). Identification of arf genes in cucurbita pepo l and analysis of expression patterns, and functional analysis of cparf22 under drought, salt stress. BMC Genomics 25, 112. doi: 10.1186/s12864-024-09992-8 38273235 PMC10809590

[B65] Zhou Y. Huang L. Liu S. Zhao M. Liu J. Lijing L. . (2023). Physiological and transcriptomic analysis of IAA-induced antioxidant defense and cell wall metabolism in postharvest mango fruit. Food Res. Int. 174, 113504. doi: 10.1016/j.foodres.2023.113504 37986499

