# Supplementary material for: Evolutionary and functional analysis of ARF and Aux/IAA gene families reveals their roles in sugar metabolism and watermelon domestication
Source: Front Plant Sci. 2026 Jun 17;17:1868206. doi: 10.3389/fpls.2026.1868206 (PMC13318979; doi:10.3389/fpls.2026.1868206)
Supplement: Supplementary file 2 [file DataSheet1.docx]

**
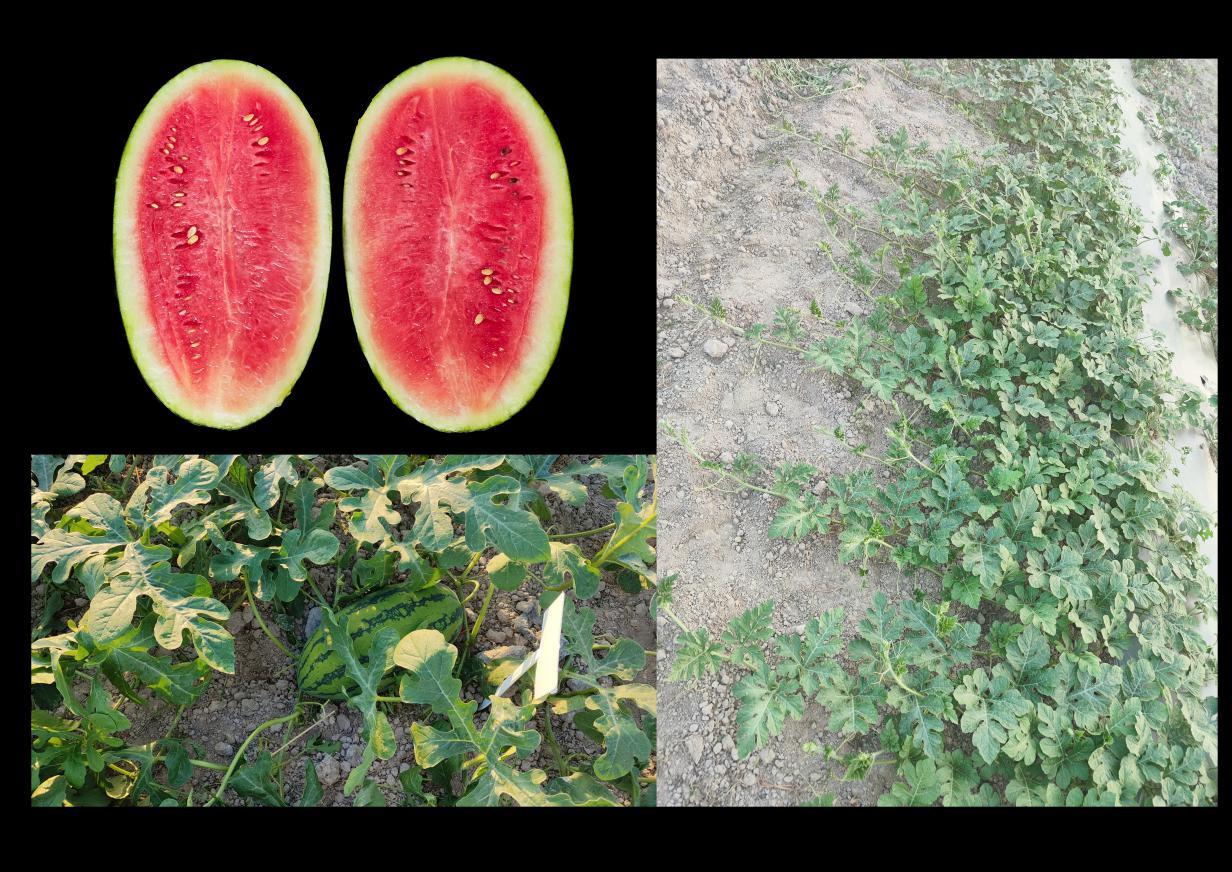
Supplementary figure S1**. Plant characteristics of 'HeiFeiCuiXuanHua'


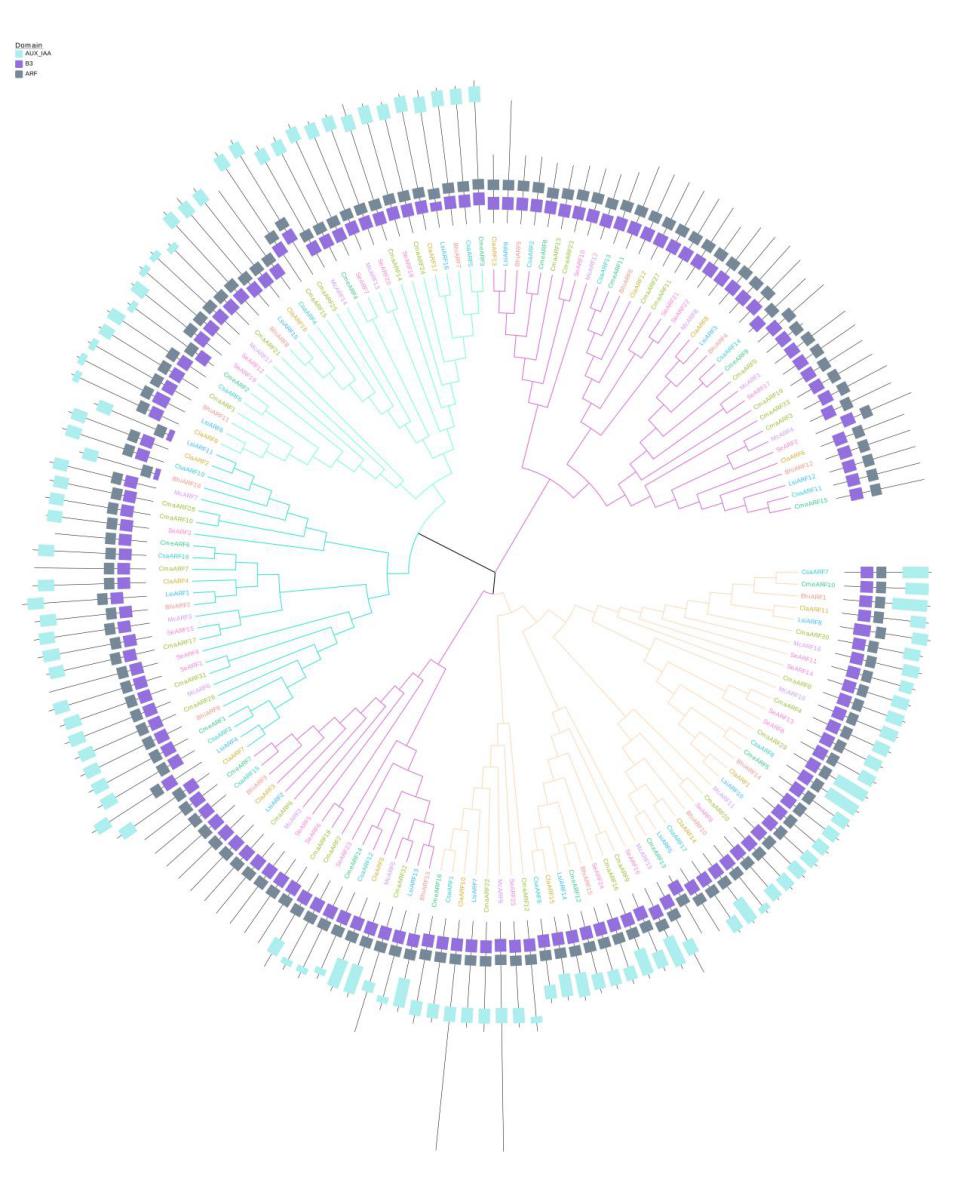
**Supplementary figure S2**. Evolutionary tree of the ARF gene family


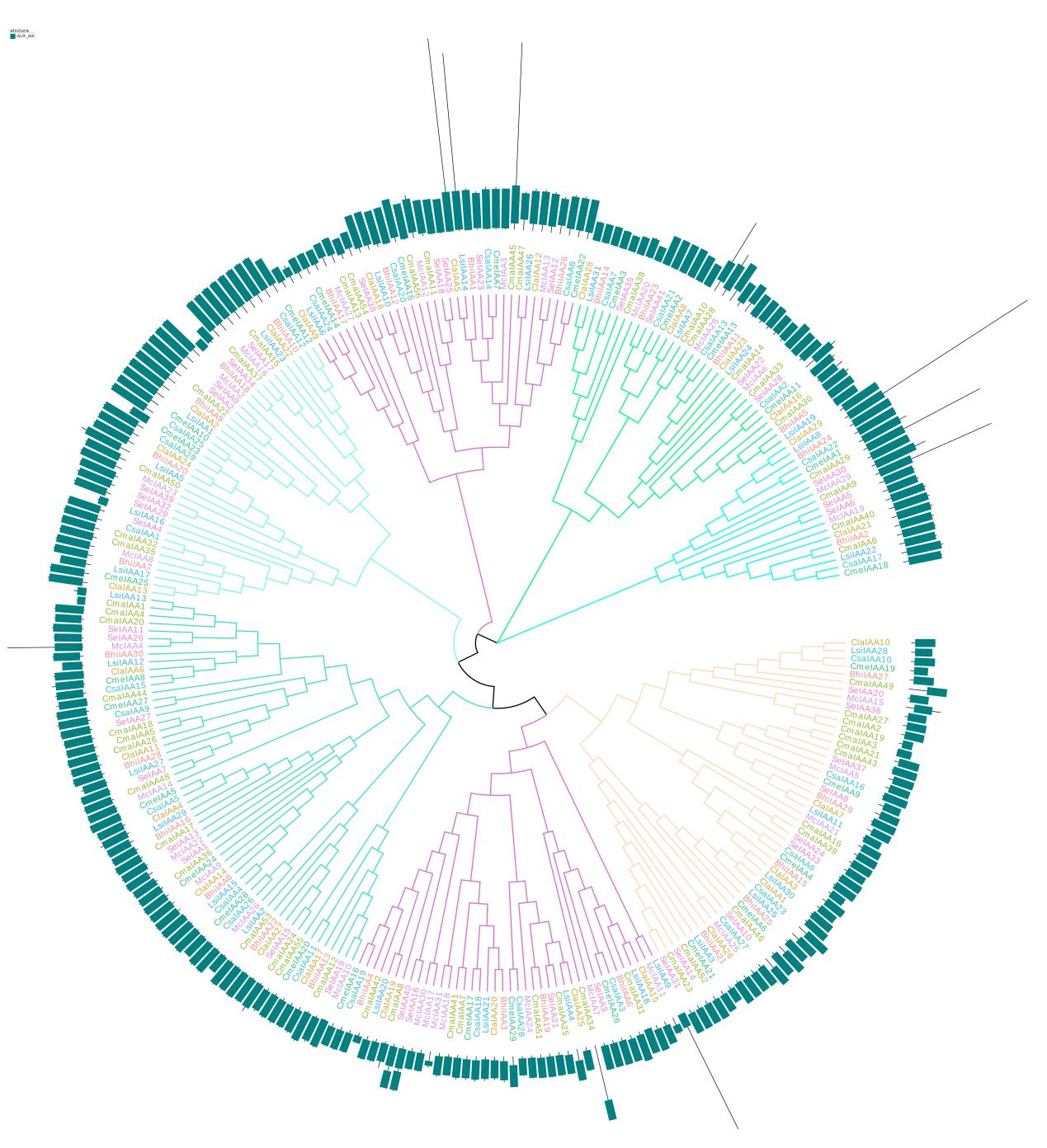


**Supplementary figure S3**. Evolutionary tree of the IAA gene family


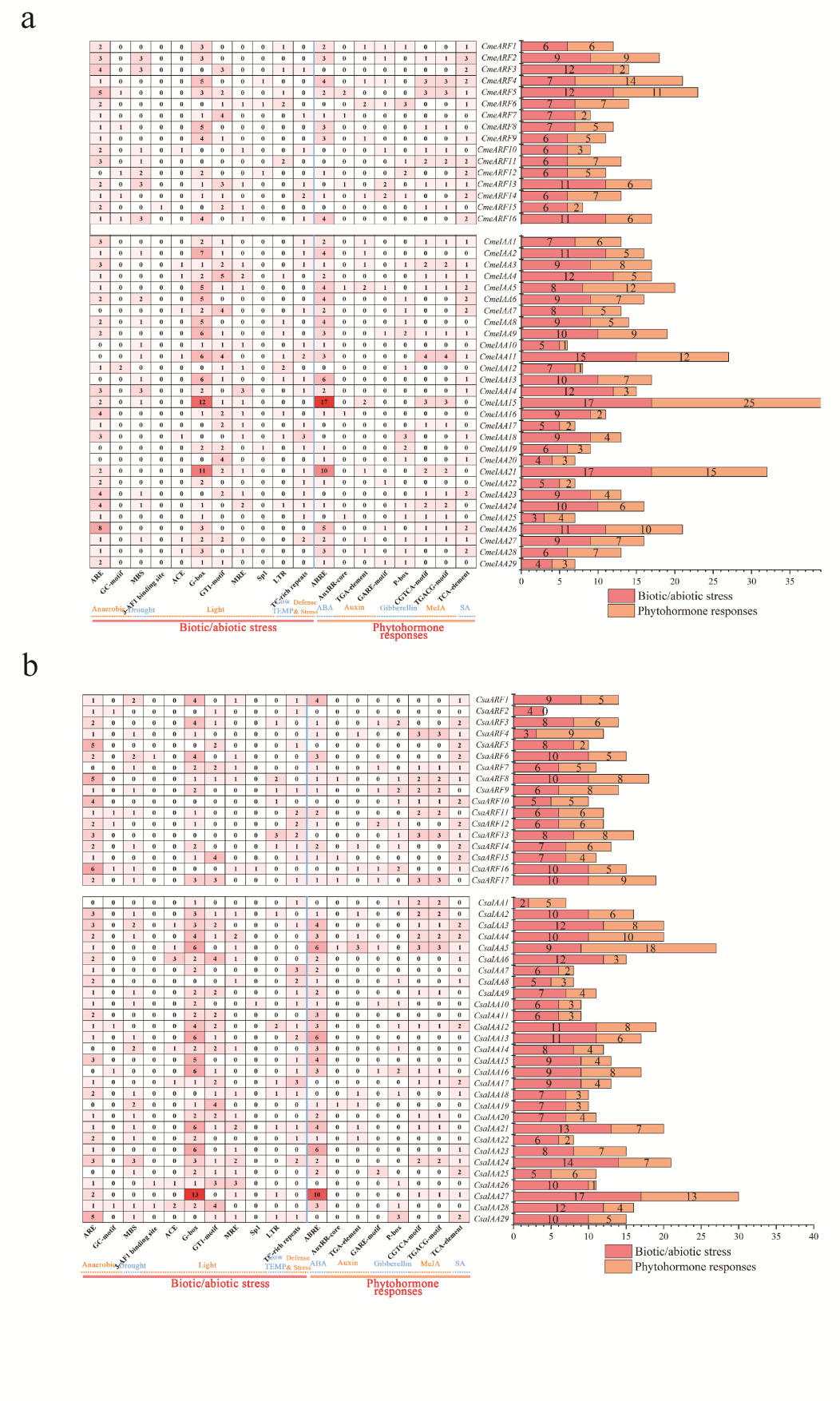


**Supplementary figure S4**. A comparative analysis of cis-regulatory elements in the promoter regions (2000 bp upstream of transcription start sites) of ARF and IAA genes from melon (a) and cucumber (b)

**
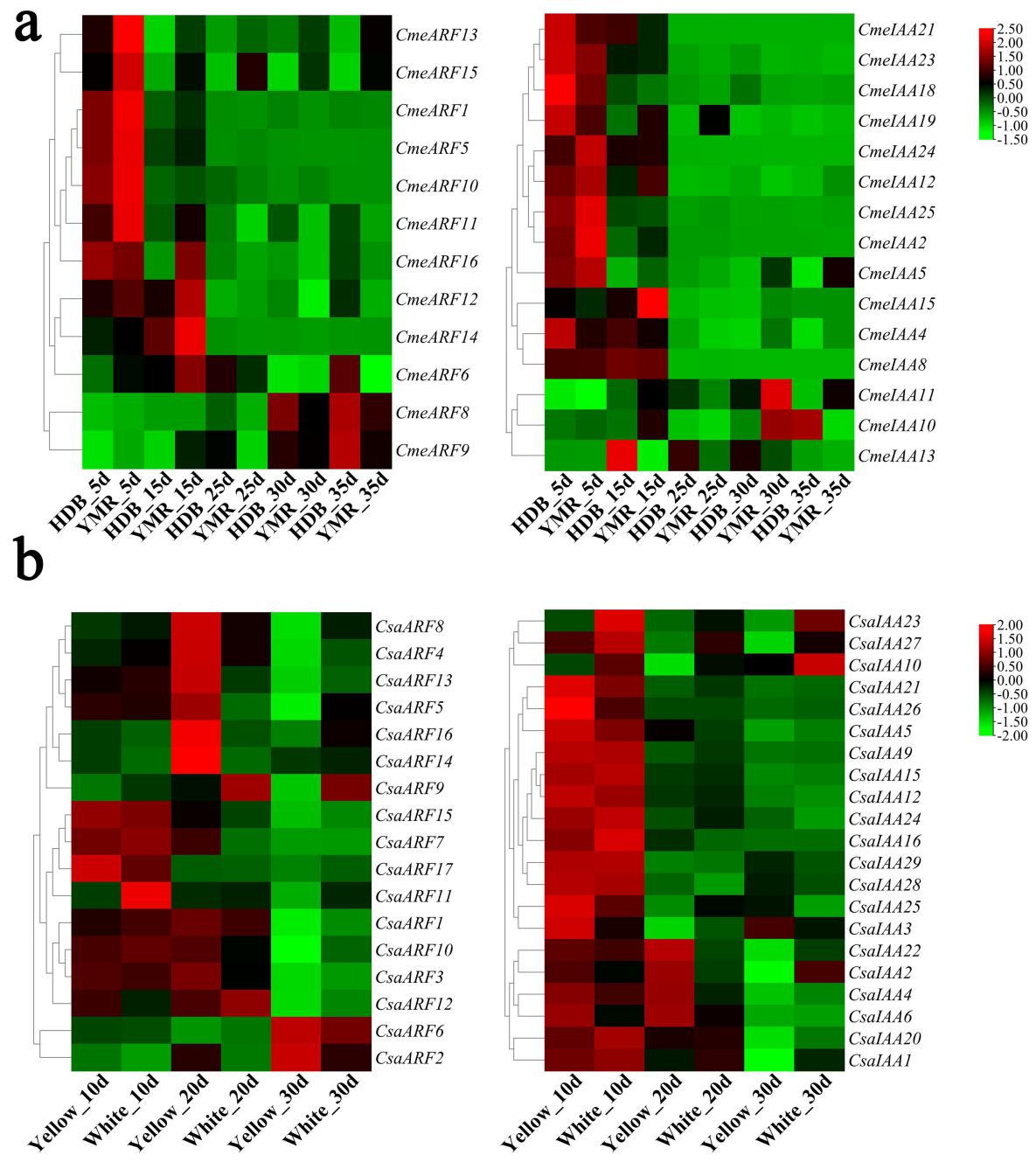
Supplementary figure S5**. Expression patterns of ARF and IAA genes during fruit development in different cucurbit accessions. (a) Melon fruits from bitter (YMR) and non-bitter (HDB) varieties at different DPP stages. (b) Cucumber fruits with yellow (Yellow) and white (White) flesh phenotypes during development.


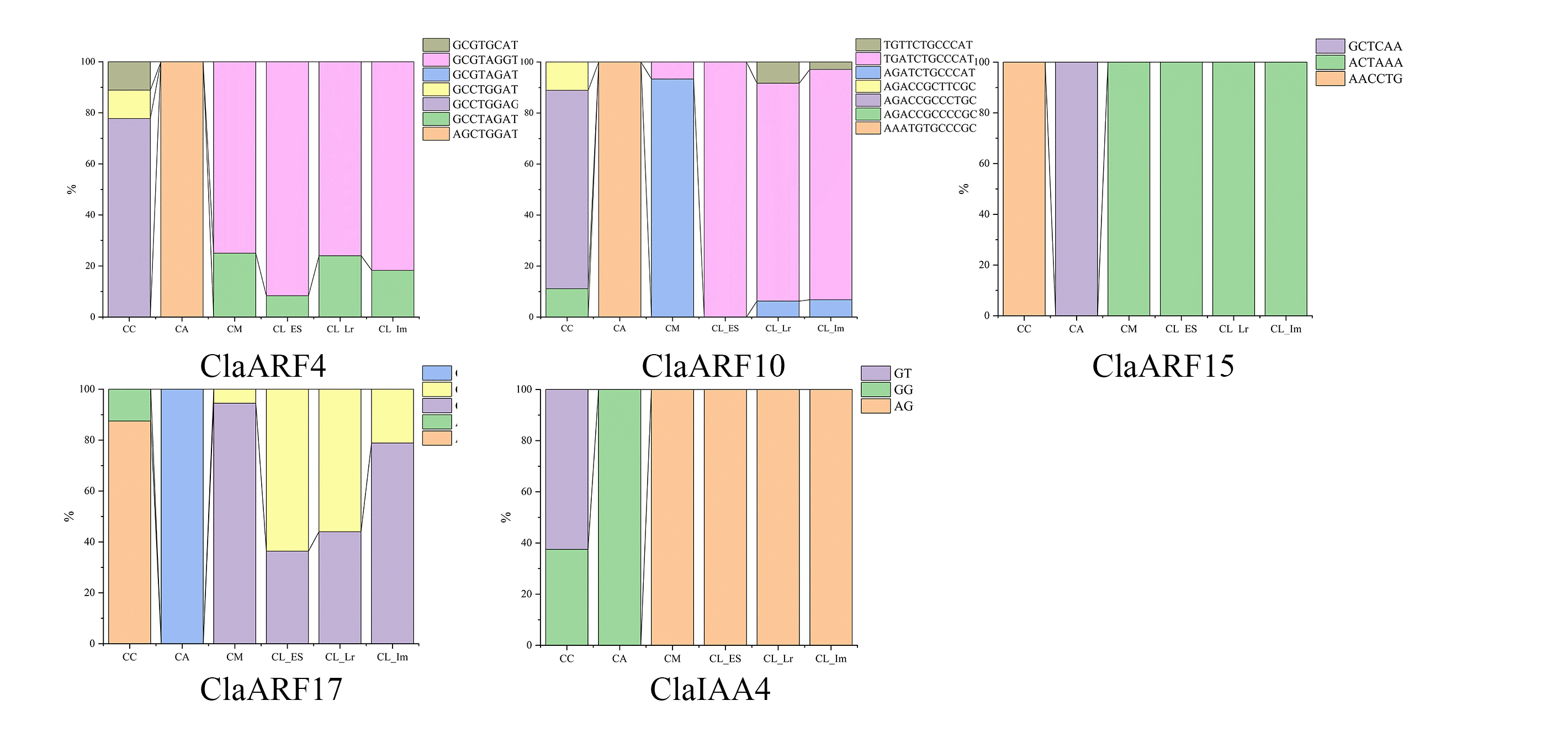


**Supplementary figure S6**. SNP Variations in *ClaARF4, ClaARF10* , *ClaARF15, ClaARF17* and *ClaIAA4* Genes During Domestication


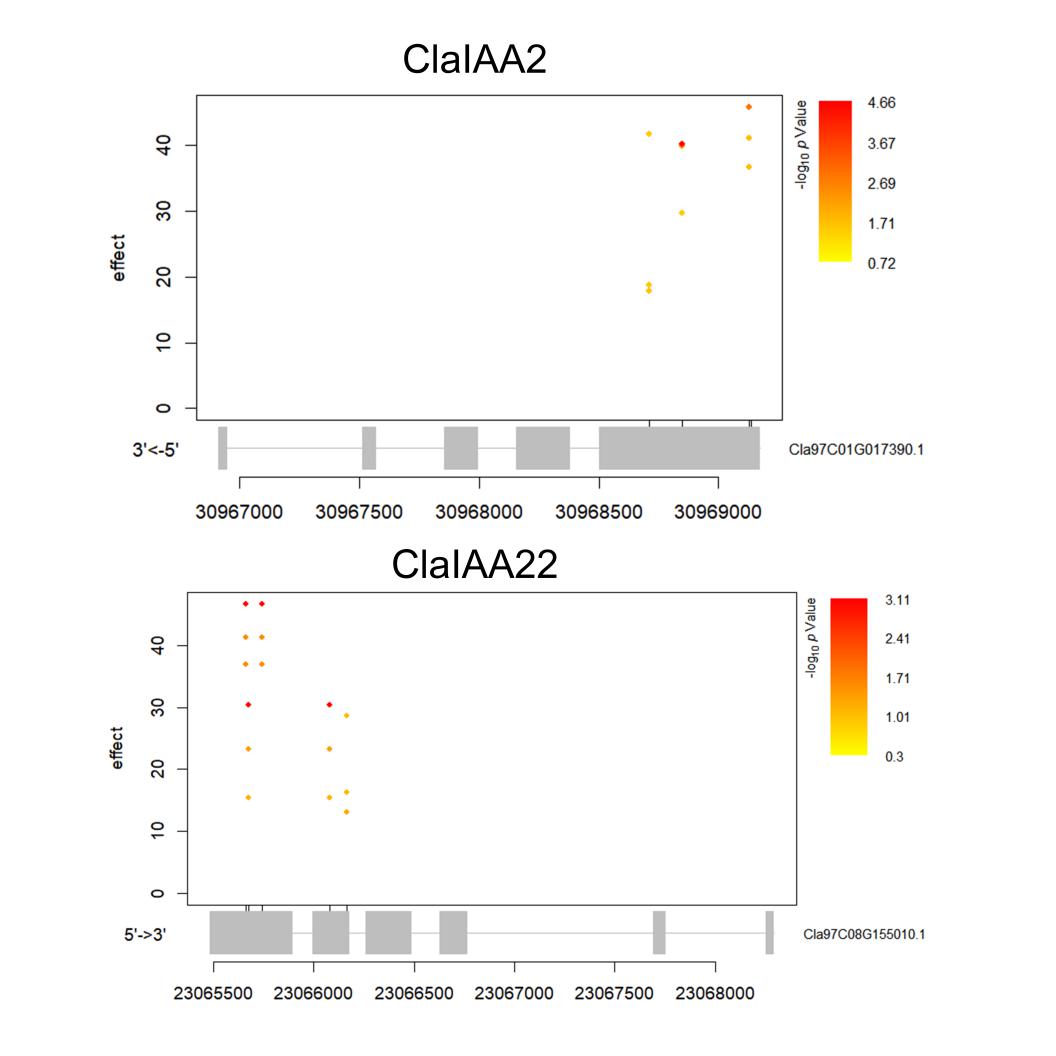
**Supplementary figure S7**. Site-specific allelic effects of *ClaIAA2*and *ClaIAA22*on soluble sugar accumulation
